# The Tandem of Liquid Chromatography and Network Pharmacology for the Chemical Profiling of Pule’an Tablets and the Prediction of Mechanism of Action in Treating Prostatitis

**DOI:** 10.3390/ph17010056

**Published:** 2023-12-28

**Authors:** Hui Zhuge, Zhiwei Ge, Jiaojiao Wang, Jianbiao Yao, Jiayu He, Yi Wang, Yingchao Wang, Yu Tang

**Affiliations:** 1 Pharmaceutical Informatics Institute, College of Pharmaceutical Sciences, Zhejiang University, Hangzhou 310058, China; 22219183@zju.edu.cn (H.Z.); zjuwangyi@zju.edu.cn (Y.W.); 2 Innovation Institute for Artificial Intelligence in Medicine, Zhejiang University, Hangzhou 310018, China; 3 Analysis Center of Agrobiology and Environment Sciences & Institute of Agrobiology and Environment Sciences, Zhejiang University, Hangzhou 310058, China; gezw@zju.edu.cn (Z.G.); wangjiaojiao2019@zju.edu.cn (J.W.); 4 Zhejiang CONBA Pharmaceutical Co., Ltd., Hangzhou 310052, China; yaojb@conbapharm.com (J.Y.); hejy2@conbapharm.com (J.H.); 5 Zhejiang Provincial Key Laboratory of Traditional Chinese Medicine Pharmaceutical Technology, Hangzhou 310052, China

**Keywords:** Pule’an tablets, prostatitis, LC-MS, network pharmacology

## Abstract

Prostatitis, a prevalent urinary tract disorder in males, has a complex etiology that leads to severe clinical discomfort. Pule’an Tablets, a classic single-component formulation primarily based on rapeseed pollen, have been clinically proven to have a beneficial therapeutic effect on both prostatitis and benign prostatic hyperplasia. However, there is currently a lack of research on the chemical composition and mechanisms of action of Pule’an Tablets in treating prostatitis. In this study, using liquid chromatography–mass spectrometry (LC-MS), a total of 53 compounds in Pule’an Tablets were identified, including flavonoids, phenylpropionamides, lipids, glucosinolates, and nucleic acids. Subsequently, through a network pharmacology analysis, potential target genes and their mechanisms of action were predicted accordingly. The results suggested that genes such as LPAR5, LPAR6, LPAR4, LPAR3, LPAR2, LPAR1, F2, ENPP2, MMP9, and TNF, along with pathways like prostate cancer, endocrine resistance, bladder cancer, and the IL-17 signaling pathway, may represent potential pathways involved in the therapeutic effects of Pule’an Tablets. This study represents the first systematic investigation into the chemical composition of Pule’an Tablets, shedding light on the potential mechanisms underlying their efficacy in treating prostatitis. These findings could serve as a valuable reference for future pharmacological research on Pule’an Tablets.

## 1. Introduction

Prostatitis is a common urological disorder and one of the most prevalent clinical conditions in males. According to an epidemiological survey, the prevalence of prostatitis ranged from 2% to 9.7% (the prevalence of different types is different), and the average prevalence was 8.2% [1]. Prostatitis has a complex etiology and leads to severe clinical discomfort, significantly affecting the quality of life for those who suffer from it. In recent years, increasing evidence suggests that prostatitis is closely associated with the occurrence, development, and prognosis of prostate cancer, calling for timely and effective therapeutic interventions [2,3]. 

Based on the pathological manifestations, the National Institutes of Health (NIH) proposed a classification for prostatitis, the most common of which is chronic prostatitis/chronic pelvic pain syndrome (CP/CPPS) (Table 1) [4,5]. Specifically, CP/CPPS is characterized by symptoms of chronic pelvic pain in the absence of a urinary tract infection. However, the etiology of CP/CPPS has not yet been established.

Common treatments for CP/CPPS include antibiotics, painkillers, alpha-blockers, physical therapy, prostate massage, lifestyle changes, and traditional Chinese medicine.

Alpha-blockers act on alpha-receptors on the surface of the smooth muscle of the prostate and bladder neck, relax the smooth muscle, and relieve the symptoms of pressure on the urethra, thereby improving urination symptoms [6]. Antibiotics are more suitable for treatment when the causative agents of prostatitis are identified. Although alpha-blockers and antibiotics have certain effects on alleviating prostatic symptoms, they may be accompanied by adverse reactions [7]. In addition, alpha-blockers and antibiotics only treat the symptoms not the underlying conditions, so it is necessary to find more effective treatments for prostatitis.

Pule’an Tablets (Qianliekang) are a classic pollen preparation capable of tonifying and strengthening the kidney. The kidney is related to the growth and reproduction of the body in Chinese medicine. In the WHO’s international standard terminologies for traditional Chinese medicine (S1), qi represents the intangible, high-mobility nutritive substance that maintains vital activities. Kidney qi deficiency syndrome refers to the decline of growth and reproductive function due to kidney qi deficiency [8]. The indications of Pule’an Tablets include kidney qi deficiency, waist and knee weakness, urine leakage or incontinence, chronic prostatitis, and prostatic hyperplasia. It has also been reported that Pule’an Tablets are effective in the treatment of type III prostatitis. Yang et al. [9] evaluated the clinical efficacy of Pule’an Tablets in treating type III prostatitis according to the National Institutes of Health Chronic Prostatitis Symptom Index (NIH-CPSI) scores, a widely used chronic prostatitis symptom scoring system. A total of 105 patients with type III prostatitis were administered with Pule’an Tablets, and the NIH-CPSI score and the number of leukocytes in the prostatic secretions were significantly decreased, which proved that the effect of Pule’an Tablets was clear in treating type III prostatitis. Clinical data also showed that Pule‘an Tablets have high efficacy in the treatment of prostate hyperplasia, with an effective cure rate of 92% [10].

Chronic inflammation plays an important role in the progression of most chronic prostatitis, evident from the benign prostatic hyperplasia where the infiltration of inflammatory cells is frequently observed in the prostate. Persistent inflammatory stimulation reshapes the prostate environment and alters the gene expression of prostate-constituting cells, which, in turn, further exacerbates inflammation, consequently leading to chronic inflammation. In animal experiments, the administration of anti-inflammatory drugs to rats having chronic prostatitis prevented the infiltration of inflammatory cells [11]. Therefore, inflammatory intervention might be an option to treat CP/CPPS. Of note, Pule’an Tablets have been reported to be effective in the treatment of chronic prostatitis and benign prostatic hyperplasia in both clinical and animal research, which can possibly be achieved by promoting local blood circulation in the prostate gland, improving microcirculation, enhancing metabolism, adjusting the physiological function of the prostate gland, and reducing inflammation. In addition, animal experiments also proved that Pule’an Tablets can significantly inhibit benign prostatic hyperplasia in rats [12]. Obviously, the therapeutic benefits of Pule’an Tablets for chronic prostatitis and prostatic hyperplasia have been widely recognized, but their chemical composition analysis and mechanism of action studies are still quite insufficient. For better understanding and more rational administration of Pule’an Tablets, there is an urgent need to carry out research on the pharmacological and material basis in the treatment of prostatitis.

Focusing on the issues mentioned above, a UPLC-Triple-TOF/MS analysis method was developed to obtain the mass spectrum data of Pule’an Tablets. A total of 53 compounds in Pule’an Tablets were identified, including 22 flavonoids, 15 phenylpropionamides, five lipids, six glucosinolates, and five nucleic acids. Subsequently, potential target genes and mechanisms of action were predicted by network pharmacology analysis. The present study may shed light on the rational use of Pule’an Tablets in prostatitis treatment and provide clues for the deep development of other botanical drugs.

## 2. Results

### 2.1. Identification of Compounds in Pule’an Tablets Based on LC-MS

**Figure 1:**
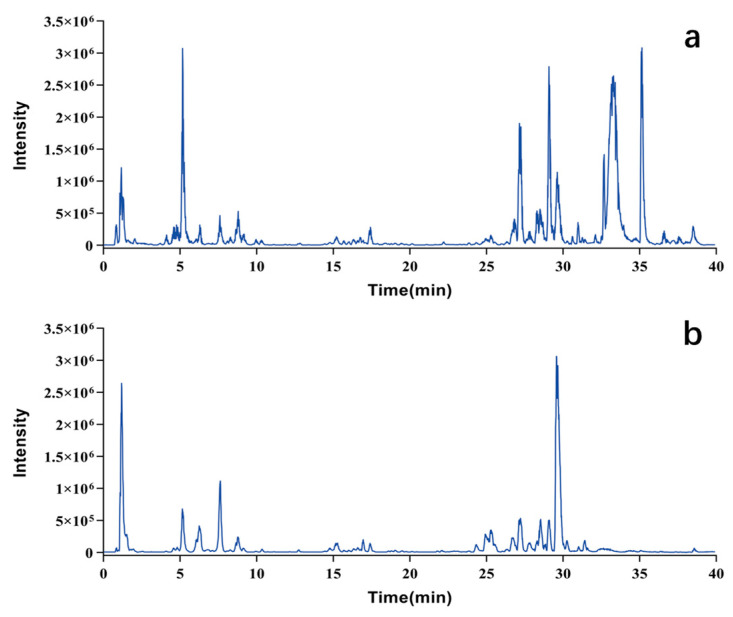
Base peak chromatograms (BPC) of Pule’an Tablets. (**a**) Positive ion mode; (**b**) negative ion mode.

Pule’an Tablets are a single preparation made from rapeseed pollen. An analytical method was established based on LC-MS technology for the chemical composition analysis of this botanical drug. Figure 1 illustrates base peak chromatograms (BPCs) of Pule’an Tablets.

**Figure 2:**
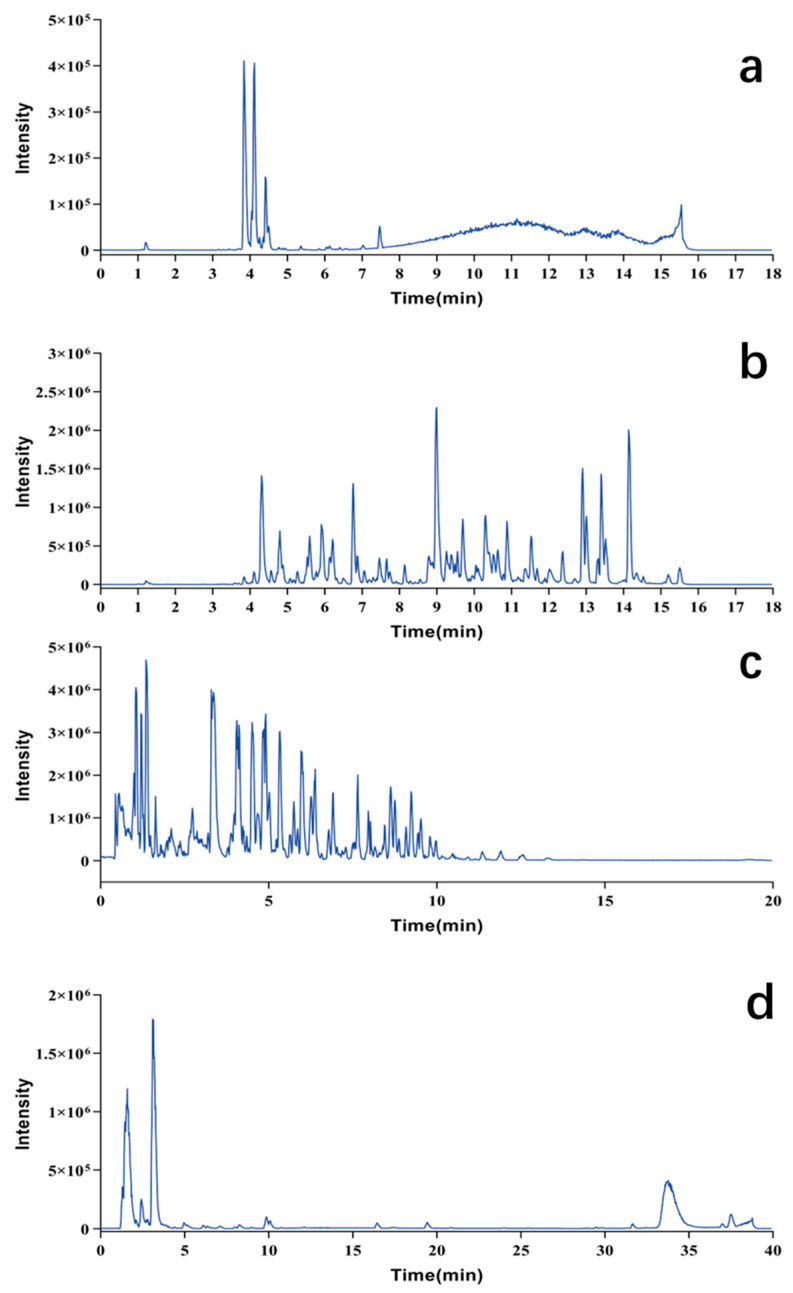
Chromatograms for chemical composition analysis of Pule’an Tablets. (**a**) Flavonoids of alcoholic extract of tablet (254 nm); (**b**) phenylpropanoids of alcoholic extract of tablet (254 nm); (**c**) lipids of alcoholic extract of tablet; (**d**) glucosinolates and nucleic acids of aqueous extract of tablet (254 nm).

The structural classes of Pule’an Tablets were preliminarily analyzed. Based on the results, the identified compounds were divided into five types, including flavonoids, phenylpropionamides, lipids, glucosinolates, and nucleic acids. Characterization methods were established for different types of chemical compounds (Section 4.4), and the five classes of identified compounds were successfully enriched and characterized in Pule’an Tablets (Figure 2).

According to the literature survey and comparison with reference materials, a total of 53 chemical compounds of Pule’an Tablets were identified. Briefly, 22 flavonoids, 15 phenylpropionamides, five lipids, six glucosinolates, and five nucleic acids were identified, as shown in Table 2. The data, in positive and negative ion modes, were analyzed by Peakview software 1.2. Please refer to Table 2 and the Supplementary Material S1 (Figures S1–S28) for more information regarding the structure elucidation.

### 2.2. Network Pharmacological Analysis of Identified Compounds

The obtained compounds were imported into SwissTargetPrediction [31,32] to predict potential targets with probability > 0. A total of 327 targets from 53 compounds of Pule’an Tablets were obtained, and duplicate targets were removed, as shown in Table 3.

**Figure 3:**
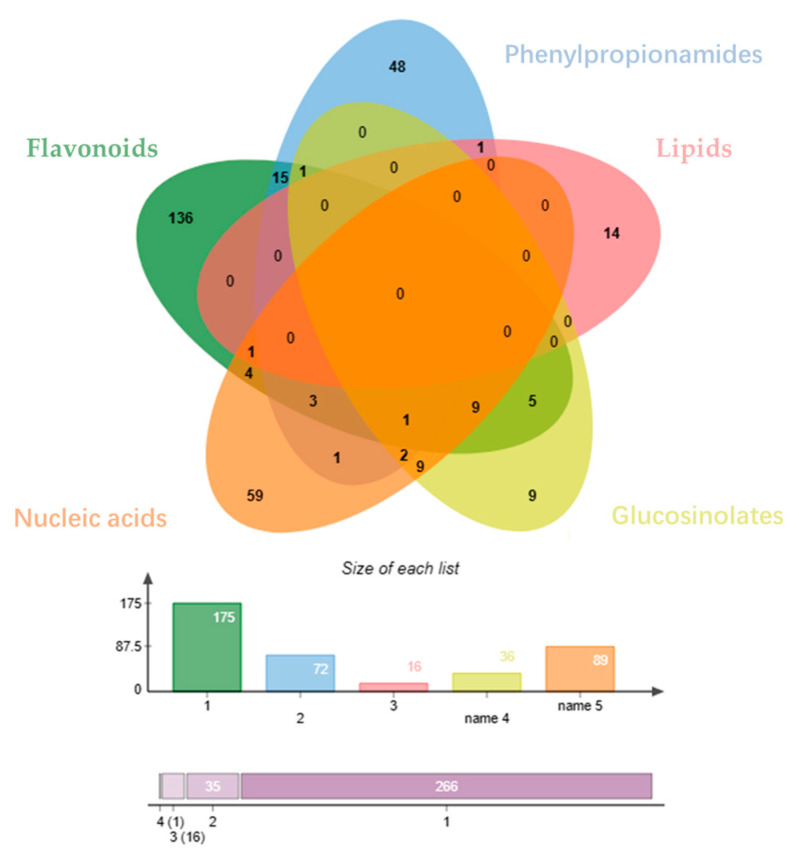
Venny diagram of targets for different compound: flavonoids (green), phenylpropionamides (blue), lipids (red), glucosinolates (yellow), and nucleic acids (orange).

**Figure 4:**
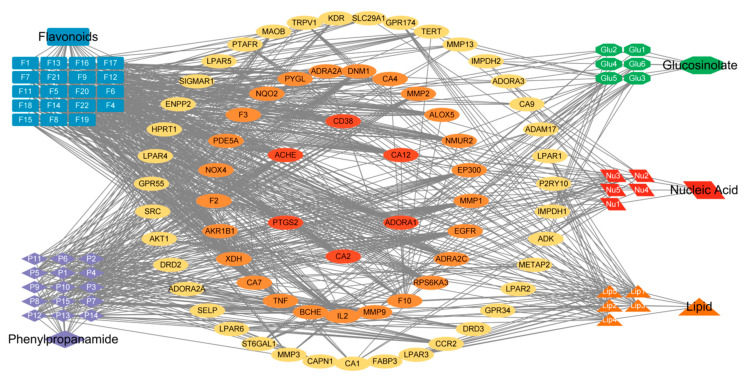
The classes-compounds-targets network of Pule’an Tablets: orange (lipid compounds), blue (flavonoids), red (glucosinolate compounds), purple (phenylpropionamide compounds), and green (nucleic acid compounds). In the middle, targets with a degree greater than 5 are screened. Use different colors to represent different degrees of gene. From the outside to the inside, the degrees of each circle are 5–14 (yellow), 15–24 (orange) and above 25 respectively(red). Please also see Supplementary Material S2 for more information about the matchups between the identified compounds and the targets.

The distribution of the Venny diagram (Figure 3) [33] shows that there is little overlap among the predicted targets of the different classes of compounds, suggesting that the different classes of compounds may synergistically play the role of action on prostatitis through different targets. By utilizing Cytoscape 3.6.1 software and the collected data, the relationship between the compounds and the targets of Pule’an Tablets was visually analyzed, and classes-compounds-targets network of Pule’an Tablets was constructed (Figure 4).

**Figure 5:**
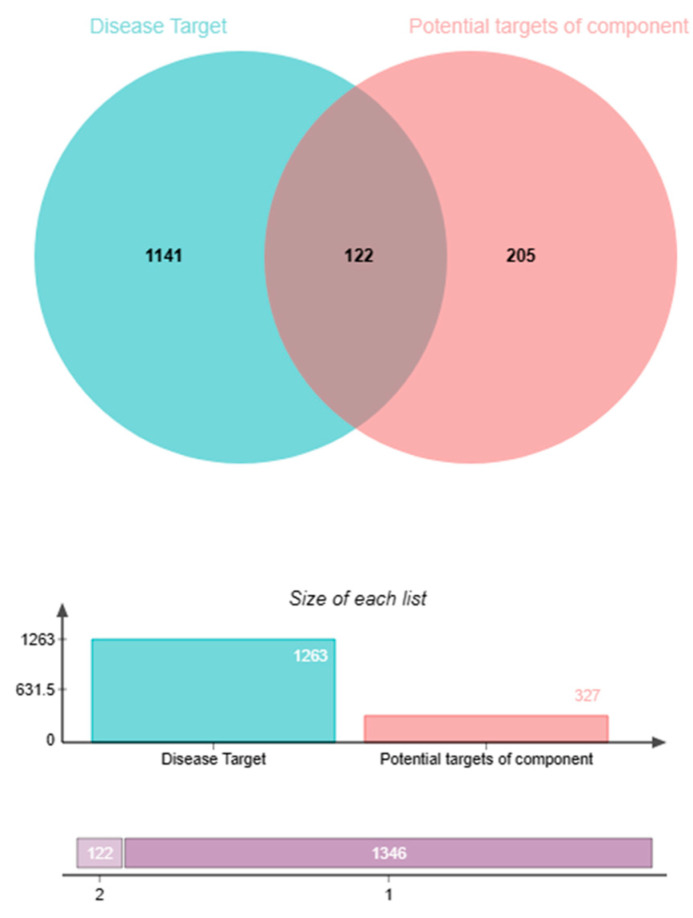
Venny diagram of the common targets of Pule’an Tablets and diseases (prostatitis, increased frequency of micturition, and prostatic hyperplasia).

Targets related to chronic prostatitis, an increased frequency of micturition, and prostatic hyperplasia were collected through GeneCards [34] and Disgenet [35]. Then, 1263 targets associated with prostatitis, an increased frequency of micturition, and prostatic hyperplasia were screened out. A total of 122 targets were consistent among prostatitis, an increased frequency of micturition, prostatic hyperplasia, and Pule’an Tablets using a Venny diagram, as shown in Figure 5.

**Figure 6:**
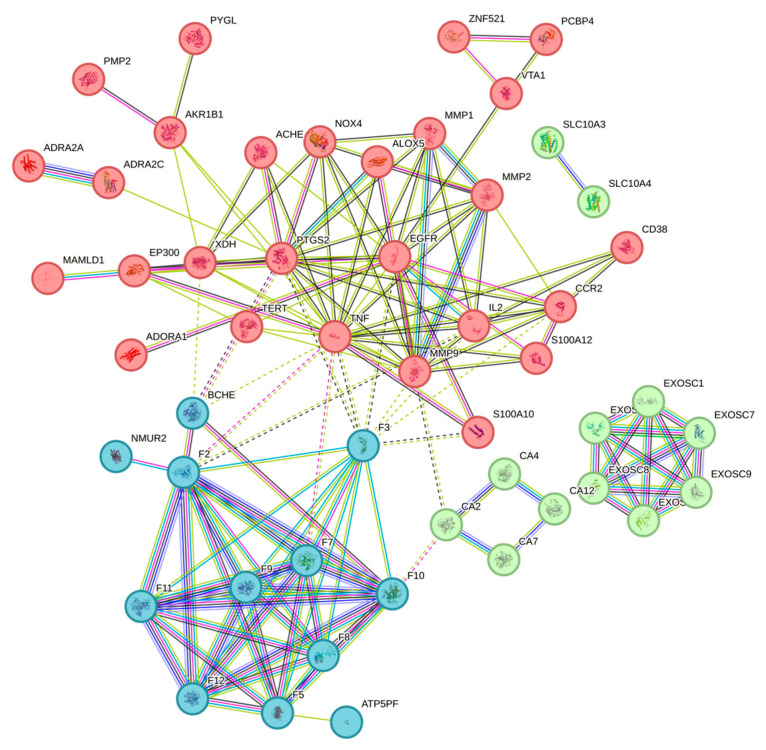
Protein-protein interaction and enrichment analysis. The same color denotes that the proteins in the cluster have similar functions.

**Figure 7:**
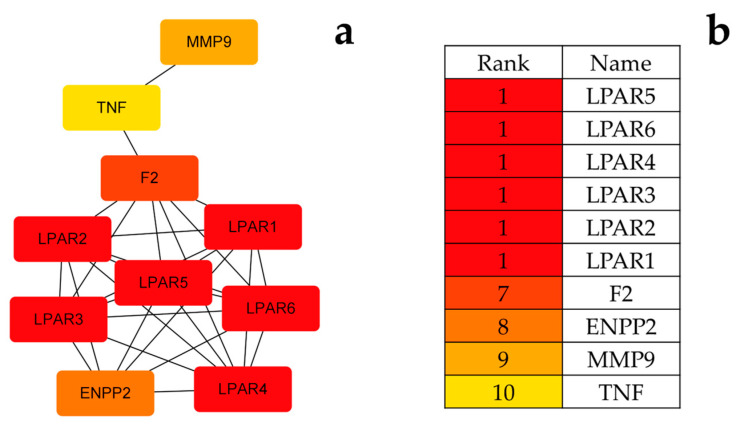
(**a**) PPI network optimized via Cytoscape 3.6.1 software; (**b**) sequencing of the top 10 target proteins.

The results suggested that these consistent targets might be related to the mechanism of disease action. The screened targets were imported into the String platform [36] and a protein-protein interaction network with a confidence of >0.7 was constructed, as shown in Figure 6. The PPI network containing 54 nodes and 102 edges was downloaded and optimized via Cytoscape 3.6.1 software. The PPI network was analyzed using the cytohubba plugin in conjunction with the MCC algorithm to sort out the top 10 genes in terms of weight share, forming 29 edges of 10 nodes, as shown in Figure 7. In order of degree, the top 10 are LPAR5, LPAR6, LPAR4, LPAR3, LPAR2, LPAR1, F2, ENPP2, MMP9, and TNF.

**Figure 8:**
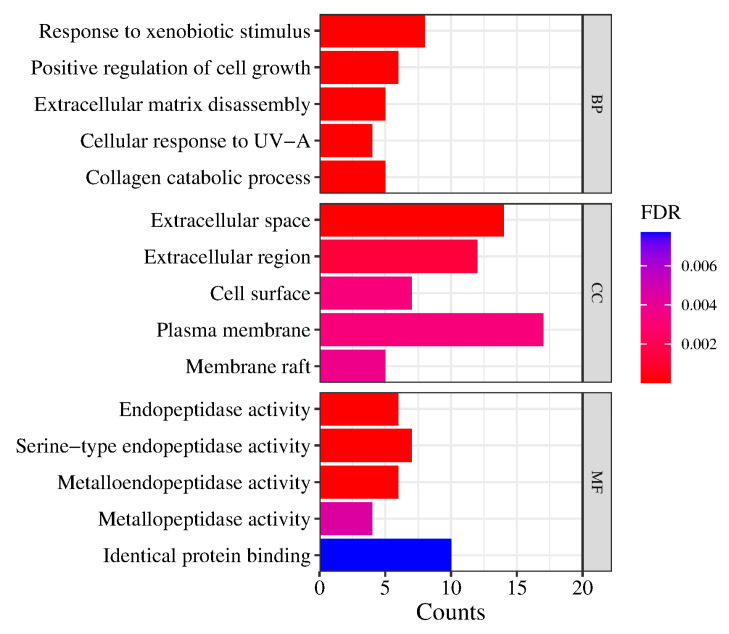
The top 5 terms from the GO-BP, GO-CC, and GO-MF enrichment analyses.

The main collected targets were imported into the DAVID database [37,38] for further bioinformatics analysis, and the potential molecular mechanisms and biological processes of the main compounds of Pule’an Tablets were explored. The GO biological process (GO-BP), the GO cellular component (GO-CC), and GO molecular function (GO-MF) term analysis were performed on the David platform. The top five terms from the GO-BP, GO-CC, and GO-MF enrichment analyses, according to the false discovery rate (FDR), are shown in Figure 8. The results showed that the chemical compounds of Pule’an Tablets have a high correlation with the GO molecular function, e.g., endopeptidase activity and serine-type endopeptidase activity. They also involve more genes in the biological processes, namely, response to xenobiotic stimulus, positive regulation of cell growth, and extracellular matrix disassembly. Due to the low FDR of these results, they can be used as key biological processes.

**Figure 9:**
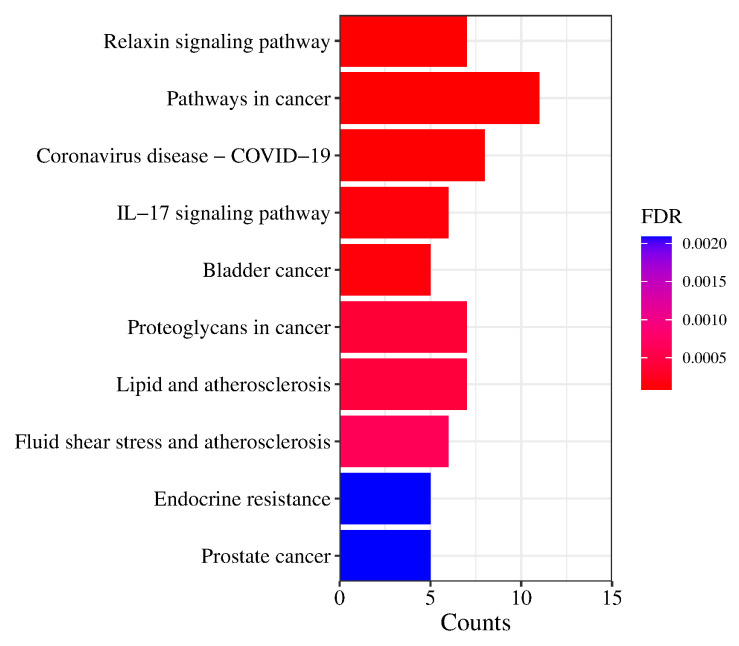
KEGG pathway analysis of potential targets of Pule’an Tablets.

For the KEGG analysis, the top 10 items are shown in Figure 9. Excluding broad-spectrum pathways such as the pathways in cancer, the results showed that prostate cancer, endocrine resistance, lipids and atherosclerosis, bladder cancer, and the IL-17 signaling pathway function in the regulation of the main components of Pule’an Tablets, which can provide reference for the subsequent study of the pharmacodynamic mechanism.

## 3. Discussion

Pule’an Tablets have been used in the clinical treatment of prostatitis and benign prostatic hyperplasia and proved to be effective. However, the chemical composition and the corresponding targets remain unknown, which impedes the pharmacological and clinical research of this botanical drug as well as the screening of lead compounds. LC-MS uses LC as the separation system and MS as the detection system, which has the advantages of the separation ability of LC and the high sensitivity and resolution of MS, andn at the same time, MS can also provide the fragmentation information for the determination of the structure [39]. Therefore, LC-MS has been widely used in the field of traditional Chinese medicine analysis in recent years [40,41].

In this study, therefore, the chemical profiles of Pule’an Tablets were characterized by the LC-MS method. It was found that the main components in Pule’an Tablets were flavonoids, phenylpropionamides, and lipids. Collectively, a total of 53 chemical components in Pule’an Tablets were identified, including 22 flavonoids, 15 phenylpropionamides, five lipids, six glucosinolates, and five nucleic acids.

Obviously, the chemical composition of Pule’an Tablets is complex, even if they are made of only a single ingredient, rapeseed pollen. To clarify the mechanism underlying the effect of Pule’an Tablets, a protein-protein interaction network and KEGG and GO pathway analyses were carried out. The results suggested that LPAR5, LPAR6, LPAR4, LPAR3, LPAR2, LPAR1, F2, ENPP2, MMP9, and TNF are potential targets for prostatitis. In addition, the main compounds in Pule’an Tablets might exert the therapeutical effect through the signaling pathways of prostate cancer, endocrine resistance, fluid shear stress and atherosclerosis, lipids and atherosclerosis, proteoglycans in cancer, bladder cancer, the IL-17 signaling pathway, the pathways in cancer, and relaxin signaling. According to the literature, MMP may play a significant role in the development of cancer [42]. Extracellular matrix (ECM) degradation plays an important role in tumor invasion and migration [43]. It is worth noting that MMP is closely related to ECM degradation and remodeling [43]. The mRNA expression of MMP1/11 in bladder cancer samples was significantly higher than that in normal bladder tissues. Although some studies showed that prostatitis is associated with prostate cancer, their relationship is still not clear. Our network pharmacological prediction results provide an idea for the application of MMP in the treatment of prostatitis and prostate cancer. Another pathway deserving of attention is the leukocyte-17 (IL-17) signaling pathway. IL-17, a pro-inflammatory cytokine that acts on various cellular targets, leads to cell activation. IL-17’s action on endothelial cells leads to inflammation and pro-coagulant activity, and it is also involved in innate and adaptive immune responses and has been proved to be associated with a variety of diseases, such as infectious diseases, autoimmune diseases, and cancer. Studies indicated that the occurrence of prostatitis may be related to inflammation and the immune reaction, and IL17A- and IFN-γ-expressing cells induce chronic pelvic pain [44]. These findings suggest that the IL17 pathway can be a potential pathway for the treatment of prostatitis.

Studies have proved that Chinese medicine has the characteristics of a multi-component, multi-target, and complex mechanism of action, and different compounds may synergistically or antagonistically act on the same target [45]. The complexity of the system of traditional Chinese medicine presents great difficulties for its in-depth study. Systemic pharmacology is a product of multidisciplinary intersection, including pharmacology, structural biology, bioinformatics, and so on. Systematic pharmacology can be used to predict pharmacokinetic and pharmacodynamic models and evaluate drug therapy mechanisms through the network analysis of databases [46,47]. The systemic pharmacology of traditional Chinese medicine provides a new idea and perspective for the study of traditional Chinese medicine. Meanwhile, it also provides ideas for us to carry out follow-up pharmacological research on Pule’an Tablets.

Collectively, these results could provide important references for subsequent pharmacological studies on the mechanism of action of Pule’an Tablets for prostatitis and also give guidance for the clinical use of Pule’an Tablets.

## 4. Materials and Methods

### 4.1. Chemicals and Reagents

Pule’an Tablets were produced by Zhejiang CONBA Pharmaceutical Co., Ltd. (Hangzhou, China). Acetonitrile and formic acid were obtained from Merk KGaA (Darmstadt, Germany). Ultrapure water was prepared by Millipore plus system.

### 4.2. Sample Preparation

#### 4.2.1. Sample Preparation for Flavonoid Analysis

First, 0.595 g of powder of crushed Pule’an Tablets was extracted by ultrasonication with 5.95 mL of 70% ethanol for half an hour at 25 °C. The extract was centrifuged, and the supernatant was collected for subsequent analysis.

#### 4.2.2. Sample Preparation for Phenylpropionamide Analysis

First, 0.599 g of powder of crushed Pule’an Tablets was extracted by ultrasonication with 5.99 mL of 70% ethanol for half an hour at 25 °C. The extract was centrifuged, and the supernatant was collected for subsequent analysis.

#### 4.2.3. Sample Preparation for Lipid Analysis

First, 0.597 g of powder of crushed Pule’an Tablets was extracted by ultrasonication with 5.97 mL of chloroform and methanol (chloroform:methanol = 1:1) for half an hour at 25 °C. The extract was centrifuged, and the supernatant was collected for subsequent analysis.

#### 4.2.4. Sample Preparation for Glucosinolate and Nucleic Acids Compounds

First, 0.595 g of powder of crushed Pule’an Tablets was extracted by ultrasonication with 5.95 mL of pure water for half an hour at 25 °C. The extract was centrifuged, and the supernatant was collected for subsequent analysis.

### 4.3. LC-MS System and Apparatus

LC-MS and MS/MS analyses were carried out on a Acquity UPLC system, which was equipped with a binary solvent delivery system, autosampler, and photodiode-array detection (DAD) system, combined with a Triple TOF 5600+ Mass Spectrometer (AB SCIEX, Singapore).

### 4.4. LC-MS Analytical Methods

#### 4.4.1. Test Conditions for Flavonoids

UPLC conditions: Sample separation was achieved using a Waters CSH-C18 (1.7 μm, 2.1 mm × 150 mm, Waters, Wexford, Ireland) at 50 °C. The flow rate was 0.35 mL/min. The injection volume was 3 μL for each run. The mobile phase was composed of 0.1% (*v*/*v*) formic acid in water (A) and 0.1% (*v*/*v*) formic acid in acetonitrile (B) with a gradient program: 0–20 min, 10–35% B; 20–35 min, 35–95% B; 35–37 min, 95% B. The detection wavelength was 254 nm.

TOF-MS conditions: Electrospray ionization source (ESI), scanning mode: positive and negative ions; GAS 1: 50 psi; GAS 2: 50 psi; curtain gas (CUS): 35 psi; desolvation gas temperature: (TEM): 550 °C (negative) and 600 °C (positive); ion source voltage (IS): −4500 V (negative) and 5500 V (positive). The mass range for data acquisition was set to *m*/*z*: 100 to 1500. The MS1 parameters were as follows: declustering potential (DP): 100 V; collision energies (CE): 10 V. Using TOF MS-Product Ion-IDA mode to record the MS2 information, collision-induced dissociation: ±40 ± 20 eV. Before injection, the mass axis correction was completed with a CDS pump to make the mass axis error less than 2 ppm.

#### 4.4.2. Test Conditions for Phenylpropionamides

UPLC conditions were in line with Section 4.4.1.

TOF-MS conditions were in line with Section 4.4.1.

#### 4.4.3. Test Conditions for Lipids

UPLC conditions: Sample separation was achieved using a Waters HSS T3-C18 (1.8 μm, 3.0 mm × 50 mm, Waters, Wexford, Ireland) at 40 °C. The flow rate was 0.5 mL/min. The injection volume was 3 μL for each run. The mobile phase was composed of 90% methanol in water (A) and Isopropanol:Acetonitrile = 1:1 (B) with a gradient program: 0–10 min, 0–11% B; 10–18 min, 100% B. The detection wavelength was 254 nm.

TOF-MS conditions were consistent with Section 4.4.1.

#### 4.4.4. Test Conditions for Glucosinolates and Nucleic Acids

UPLC conditions: Sample separation was achieved using a ThermoFisher HYPERCARB (2.1 mm × 100 mm, Los Angeles, CA, USA) at 50 °C. The flow rate was 0.25 mL/min. The injection volume was 3 μL for each run. The mobile phase was composed of 0.3% ammonia and 0.1% ammonium acetate in water (A) acetonitrile (B) with a gradient program: 0–2 min, 2–7% B; 2–25 min, 7–25% B; 25–37 min, 25–95% B. The detection wavelength was 254 nm.

TOF-MS conditions were in line with Section 4.4.1.

### 4.5. Targeted Network Pharmacology Analysis

The components obtained by inference or prediction from LC-MS analysis were imported into SwissTargetPrediction to predict the corresponding targets, and targets with a possibility > 0 were collected for prediction. Disease targets were acquired in the DisGeNet database and Genecards database. Chronic prostatitis, prostate hyperplasia, and urinary-frequency disease genes were searched, and genes with a relevance score greater than 10 were selected, while duplicate targets were screened out. Cytoscape 3.6.1 was used for visualization analysis to screen the gene targets with degree > 5. PPI analysis was performed through the STRING platform. Cytoscape software (Version 3.6.1) was used to further analyze the PPI network, and the cytoHubba plug-in was applied to screen the most core targets in the network according to the MCC algorithm. GO analysis and KEGG analysis were performed using the DAVID platform.

## 5. Conclusions

In conclusion, while the biological activity and potential phytotherapeutic role of the compounds require further study, the present work systematically investigated the chemical composition of Pule’an Tablets. A total of 53 compounds encompassing five structural classes were identified. In addition, potential pathways and bioactive compounds involved in the therapeutic effects of this botanical drug were studied through a network pharmacology analysis. Collectively, the findings based on both wet-and dry-lab experiments in this research could shed light on the potential mechanisms underlying their efficacy in treating prostatitis, can serve as a valuable reference for future pharmacological research on Pule’an Tablets, and are of guiding significance for the further screening and subsequent development of active substances in other botanical drugs.

## Figures and Tables

**Table 1:** Classification of prostatitis syndromes.

Type	Name
I	Acute bacterial prostatitis (ABP)
II	Chronic bacterial prostatitis (CBP)
III	Chronic prostatitis/chronic pelvic pain syndrome (CP/CPPS)
III-A	Inflammatory subtype
III-B	Non-inflammatory subtype
IV	Asymptomatic inflammatory prostatitis (histological prostatitis)

**Table 2:** The identified compounds of Pule’An Tablets based on UPLC coupled with quadrupole TOF-MS (UPLC-Q-TOF-MS).

No.	t_R_ (min)	Name	Molecular Formula	*m*/*z*	Fragment Ions	Class	Reference
1	6.30	3,7-Di-*O*-glucosylkaempferol ^b^	C_27_H_30_O_16_	609.1446[M − H]^−^	446.0866, 283.0241	Flavonoids	[13]
2	7.61	Kaempferol-3-*O*-sophoroside ^a^	C_27_H_30_O_16_	609.1445[M − H]^−^	429.0832, 285.0396	Flavonoids	[14]
3	9.25	Isorhamnetin 3-*O*-*β*-*D*-xylopyranosyl (1→2)-*β*-*D*-glucopyranoside ^c^	C_27_H_30_O_16_	609.1443[M − H]^−^	315.0507	Flavonoids	
4	6.24	Quercetin-*3*-*O*-*D*-glucoside-*7*-*O*-*D*-glucoside ^b^	C_27_H_30_O_17_	625.1397[M − H]^−^	463.0889, 301.0346	Flavonoids	[15]
5	10.35	Astragalin ^a^	C_21_H_20_O_11_	447.0917[M − H]^−^	285.0403	Flavonoids	
6	11.14	Kaempferol 7-*O*-glucoside ^b^	C_21_H_20_O_11_	447.0919[M − H]^−^	285.0400	Flavonoids	[15]
7	12.74	Kaempferol 3-rhamnoside ^b^	C_21_H_20_O_10_	431.0971[M − H]^−^	285.0399	Flavonoids	[16]
8	8.66	Isoquercetin ^a^	C_21_H_20_O_12_	463.0864[M − H]^−^	301.0365	Flavonoids	
9	6.71	Isorhamnetin 3,7-*O*-di-*β*-*D*-glucopyranoside ^c^	C_28_H_32_O_18_	639.1556[M − H]^−^	476.0998, 313.0358	Flavonoids	
10	7.11	Isorhamnetin 3-*O*-sophoroside ^c^	C_28_H_32_O_18_	639.1554[M − H]^−^	315.0505	Flavonoids	
11	6.97	Quercetin 3-*O*-*β*-*D*-glucopyranosyl-(2→1)-O-*β*-*D*-xylopyranoside ^c^	C_26_H_28_O_16_	595.1284[M − H]^−^	301.0351	Flavonoids	
12	12.14	Kaempferol 3-*O*-(6″-O-malonyl) glucoside ^c^	C_24_H_22_O_14_	533.0919[M − H]^−^	285.0410	Flavonoids	
13	13.04	Isorhamnetin-3-*O*-rhamnoside ^b^	C_22_H_22_O_11_	461.1072[M − H]^−^	315.0553	Flavonoids	[17]
14	10.73	Isorhamnetin-3-*O*-glucoside ^b^	C_22_H_22_O_12_	477.1022[M − H]^−^	315.0503	Flavonoids	[18]
15	8.13	Kaempferol 3-*O*-*α*-*L*-arabinopyranosyl-(1‴→6″)-*β*-*D*-glucopyranoside ^c^	C_26_H_28_O_15_	579.1337[M − H]^−^	285.0396	Flavonoids	
16	8.87	3-*O*-[*β*-*D*-xylopyranosyl-(1→2)-*β*-*D*-glucopyranosyl]-kaempferol ^c^	C_26_H_28_O_15_	579.1332[M − H]^−^	285.0402	Flavonoids	
17	8.84	Kaempferol-3-rutinoside ^b^	C_27_H_30_O_15_	593.1495[M − H]^−^	285.0394	Flavonoids	[19]
18	9.03	Isorhamnetin 3-*O*-neohesperidoside ^b^	C_28_H_32_O_16_	623.1670[M − H]^−^	315.0517	Flavonoids	[20]
19	9.11	Kaempferol 3-*O*-*β*-*D*-(2-*O*-*β*-*D*-6-*O*-acetylglucosyl)-glucopyranoside ^c^	C_29_H_32_O_17_	651.1540[M − H]^−^	285.0373	Flavonoids	
20	16.95	Naringenin ^a^	C_15_H_12_O_5_	271.0611[M − H]^−^	151.0039	Flavonoids	
21	15.27	Quercetol ^a^	C_15_H_10_O_6_	301.0334[M − H]^−^	151.0035, 178.9968	Flavonoids	
22	19.58	Isorhamnetin ^b^	C_16_H_12_O_7_	315.0500[M − H]^−^	151.0048, 300.0267	Flavonoids	[16]
23	5.14	N1, N10-bis (p-coumaroyl) spermidine ^c^	C_25_H_31_N_3_O_4_	436.2228[M − H]^−^	119.0503, 145.0289	Phenylpropionamides	
24	8.08	N5, N14-dicoumaroyl-N1-caffeoylspermidine ^c^	C_37_H_44_N_4_O_7_	655.3122[M − H]^−^	135.0458	Phenylpropionamides	
25	8.67	N10, N14-dicoumaroyl-N1-caffeoylspermidine ^c^	C_37_H_44_N_4_O_7_	655.3120[M − H]^−^	135.0451	Phenylpropionamides	
26	15.71	N1, N5, N10-(Z)-tri-p-coumaroylspermidine^c^	C_34_H_37_N_3_O_6_	582.2594[M − H]^−^	119.0507, 145.0300	Phenylpropionamides	
27	16.35	N1, N5-(Z)-N10-(E)-tri-p-coumaroylspermidine ^c^	C_34_H_37_N_3_O_6_	582.2590[M − H]^−^	119.0507, 145.0285	Phenylpropionamides	
28	16.78	N1, N0-(E)-N5-(Z)-tri-p-coumaroylspermidine ^c^	C_34_H_37_N_3_O_6_	582.2589[M − H]^−^	119.0521, 145.0297	Phenylpropionamides	
29	17.41	N1, N5, N10-tri-p-coumaroylspermidine	C_34_H_37_N_3_O_6_	582.2593[M − H]^−^	119.0503, 145.0286	Phenylpropionamides	
30	17.59	N5, N10-dicoumaroyl-N1, N14-dicaffeoylspermidine ^c^	C_46_H_50_N_4_O_10_	817.3430[M − H]^−^	119.1450	Phenylpropionamides	
31	16.03	N10-caffeoyl-N1, N5-di-p-coumaroylspermidine ^c^	C_34_H_37_N_3_O_7_	598.2543[M − H]^−^	119.0514, 161.0236	Phenylpropionamides	
32	18.61	N1-N5-N10-N14-(Z)-tetra-p-coumaroylspermine ^c^	C_46_H_50_N_4_O_8_	785.3542[M − H]^−^	145.0292	Phenylpropionamides	
33	19.06	N1-N14-(Z)-N5-N10(E)-tetra-p-coumaroylspermine ^c^	C_46_H_50_N_4_O_8_	785.3544[M − H]^−^	145.0291	Phenylpropionamides	
34	19.49	N1-N14-(E)-N5-N10(Z)-tetra-p-coumaroylspermine ^c^	C_46_H_50_N_4_O_8_	785.3540[M − H]^−^	119, 145.0309	Phenylpropionamides	
35	20.14	N1, N5, N10, N14-tetra-p-coumaroylspermine ^c^	C_46_H_50_N_4_O_8_	785.3543[M − H]^−^	119, 145.0286	Phenylpropionamides	
36	12.95	N1, N5, N10-(E)-tricaffeoylspermidine	C_34_H_37_N_3_O_9_	630.2441[M − H]^−^	135.0441, 161.0240	Phenylpropionamides	
37	12.85	N, N′-di(coumaroyl)putrescine ^c^	C_22_H_24_N_2_O_4_	379.1653[M − H]^−^	119.0504, 145.0295	Phenylpropionamides	
38	4.12	1,2-Dilinolenoyl-sn-glycero-3-phosphocholine ^c^	C_44_H_76_NO_8_P	778.5345[M − H]^+^	184.0714	Lipids	
39	4.52	PC-C18:3/C18:2 ^c^	C_44_H_78_NO_8_P	780.5506[M − H]^+^	184.0728	Lipids	
40	4.88	1-Palmitoyl-2-linoleoyl-3-sn-phosphatidylcholine ^c^	C_42_H_78_NO_8_P	756.5507[M − H]^+^	184.0719	Lipids	
41	5.04	1,2-Dilinoleoyl-sn-glycero-3-phosphocholine ^c^	C_44_H_80_NO_8_P	782.5666[M − H]^+^	184.0736	Lipids	
42	5.33	Lecithin ^b^	C_42_H_80_NO_8_P	758.5665[M − H]^+^	184.0727	Lipids	[21]
43	3.58	5′-Uridylic acid ^b^	C_9_H_13_N_2_O_9_P	323.0290[M − H]^−^	280.0220, 211.0015	Nucleic acids	[22]
44	9.59	Uridine ^b^	C_9_H_12_N_2_O_6_	243.0635[M − H]^−^	200.0576, 152.0353, 110.0263	Nucleic acids	[23]
45	9.78	5′-Adenosine monophosphate ^b^	C_10_H_14_N_5_O_7_P	346.0563[M − H]^−^	280, 211.0013, 96.9719, 78.9632	Nucleic acids	[24]
46	16.18	Cyclic guanosine 3′,5′-monophosphate ^b^	C_10_H_12_N_5_O_7_P	344.0410[M − H]^−^	150.0427, 133.0164	Nucleic acids	[25]
47	19.15	Adenosine 3′,5′-cyclic monophosphate ^b^	C_10_H_12_N_5_O_6_P	328.0459[M − H]^−^	134.0482	Nucleic acids	[25]
48	5.63	Sinigrin ^a^	C_10_H_17_NO_9_S_2_	358.0276[M − H]^−^	96.9613	Glucosinolates	[26]
49	6.60	Progoitrin ^c^	C_11_H_19_NO_10_S_2_	388.0386[M − H]^−^	96.9625	Glucosinolates	
50	8.92	Gluconapin ^b^	C_11_H_19_NO_9_S_2_	372.0432[M − H]^−^	96.9634	Glucosinolates	[27]
51	9.3	Glucoraphanin ^b^	C_12_H_23_NO_10_S_3_	436.0415[M − H]^−^	96.9621	Glucosinolates	[28]
52	11.83	Glucoalyssin ^b^	C_13_H_25_NO_10_S_3_	450.0570[M − H]^−^	386.0626,96.9633	Glucosinolates	[29]
53	20.6	Gluconasturtiin ^b^	C_15_H_21_NO_9_S_2_	422.0588[M − H]^−^	96.9623	Glucosinolates	[30]

^a^ Structure confirmed by comparison with authentic standards. ^b^ Structure confirmed by the reference. ^c^ Identified structure needs further verification.

**Table 3:** Number of predicted targets for different kinds of chemical compounds.

Data Type	Chemical Compound Type					Total
	Phenylpropionamides	Nucleic Acids	Glucosinolates	Flavonoids	Lipids	
Chemical composition	15	6	5	22	5	53
Target	72	89	36	175	16	327

## Data Availability

Data are available within the article and Supplementary Materials.

## Supplementary Materials

The following supporting information can be downloaded at https://www.mdpi.com/article/10.3390/ph17010056/s1, Supplementary Material S1: Identified structure in Pule’an Tablets. Supplementary Material S2: The identified compounds of Pule’an Tablets and corresponding targets.
