# Supplementary material for: The Tandem of Liquid Chromatography and Network Pharmacology for the Chemical Profiling of Pule’an Tablets and the Prediction of Mechanism of Action in Treating Prostatitis
Source: Pharmaceuticals (Basel). 2023 Dec 28;17(1):56. doi: 10.3390/ph17010056 (PMC10821205; doi:10.3390/ph17010056)
Supplement: Supplementary file 1 [file pharmaceuticals-17-00056-s001.zip › Supplementary material S1.pdf]

Supplementary Material

The Tandem of Liquid Chromatography and Network Pharmacology for the Chemical Profiling of Pule'an Tablets and the Prediction of Mechanism of Action in Treating Prostatitis

Hui Zhuge [1,2], Zhiwei Ge [3], Jiaojiao Wang [3], Jianbiao Yao [4,5], Jiayu He [4,5], Yi Wang [1], Yingchao Wang [1,2,*] and Yu Tang[1,2,*]

1. Pharmaceutical Informatics Institute, College of Pharmaceutical Sciences, Zhejiang University, Hangzhou 310058, China; 22219183@zju.edu.cn (H.Z.); zjuwangyi@zju.edu.cn (Y.W.)
2. Innovation Institute for Artificial Intelligence in Medicine of Zhejiang University, Hangzhou 310018, China; 22219183@zju.edu.cn (H.Z.)
3. Analysis Center of Agrobiology and Environment Sciences& Institute of Agrobiology and Environment Sciences, Zhejiang University, Hangzhou 310058, China; gezw@zju.edu.cn (Z.G.); wangjiaojiao2019@zju.edu.cn (J.W.)
4. Zhejiang CONBA Pharmaceutical Co., Ltd, Hangzhou 310052, China; yaojb@conbapharm.com (J.Y.); hejy2@conbapharm.com (J.H.)
5. Zhejiang Provincial Key Laboratory of Traditional Chinese Medicine Pharmaceutical Technology, Hangzhou 310052, China; yaojb@conbapharm.com (J.Y.); hejy2@conbapharm.com (J.H.)
Correspondence: wangyingchao@zju.edu.cn (Y. W.); yutang@zju.edu.cn (Y. T.)

Supplementary results

1. WHO international standard terminologies on traditional Chinese medicine

https://www.who.int/publications/i/item/9789240042322

2. Identifiction of compounds

The peak of Compound 3 peak was appeared at 9.27 min with the molecular ion at *m/z* 609.1443 $[M-H]^-$, and the molecular formula fitted according to the MS^1 results was $C_{27}H_{30}O_{16}$. According to the fragment of 315.0507 $[M-H]^-$ in MS^2 spectra, the presence of glucose structure and xylose structure of the compound was hypothesized. Parent nuclide of the compound is isorhamnetin. After searching of the Scifinder and Reaxys databases, compound 3 was deduced as Isorhamnetin 3-*O*-*β*-*D*-xylopyranosyl (1→2)-*β*-*D*-glucopyranosidec, the spectra of MS^1, MS^2 and the possible structural formula of this compound are shown in the following figure.

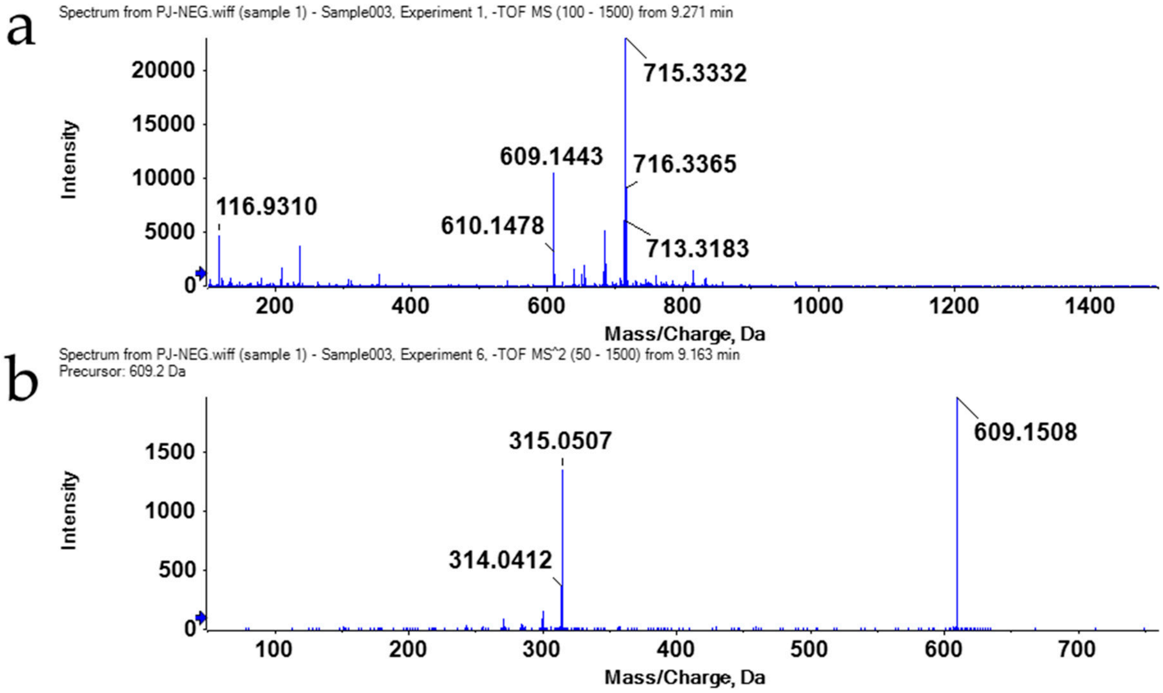

Figure S1. Compound 3. (a) Primary mass spectrum;(b) Secondary mass spectrum.

The peak of Compound 9 was appeared at 6.71 min with the molecular ion at *m*/*z* 639.1556 $[M-H]^-$, and the molecular formula fitted according to the MS^1 results was $C_{28}H_{32}O_{18}$. According to the fragment of 313.0358 $[M-H]^-$, 476.0998 $[M-H]^-$ in MS^2 spectra, the presence of glucose structure of the compound was hypothesized. Parent nuclide of the compound is isorhamnetin. After searching of the Scifinder and Reaxys databases, compound 9 was deduced as isorhamnetin 3-*O*-sophoroside, the spectra of MS^1, MS^2 and the possible structural formula of this compound are shown in the following figure.

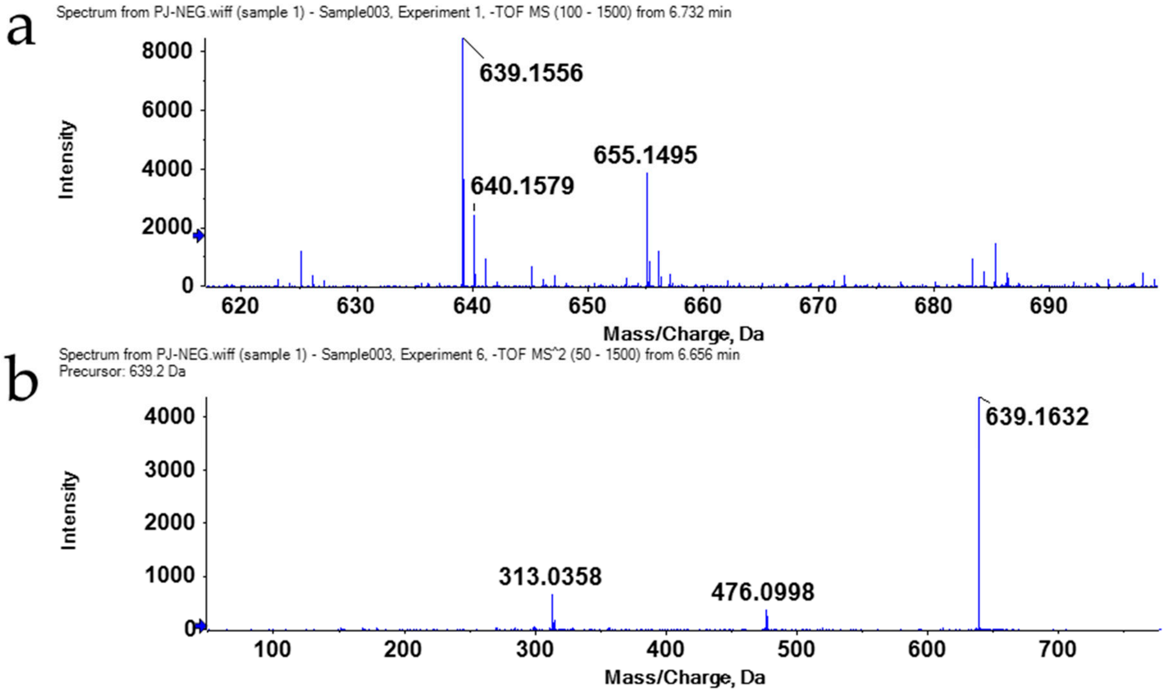

Figure S2. Compound 9 (a) Primary mass spectrum; (b) Secondary mass spectrum.

The peak of Compound 10 was appeared at 7.11 min with the molecular ion at *m*/*z* 639.1554 $[M-H]^-$, and the molecular formula fitted according to the MS^1 results was $C_{28}H_{32}O_{18}$. According to the fragment of 315.0505 $[M-H]^-$ in MS^2 spectra, the presence of glucose structure of the compound was hypothesized. Parent nuclide of the compound is isorhamnetin. After searching of the Scifinder and Reaxys databases, compound 10 was deduced as Isorhamnetin 3-*O*-sophoroside, the spectra of MS^1, MS^2 and the possible structural formula of this compound are shown in the following figure.

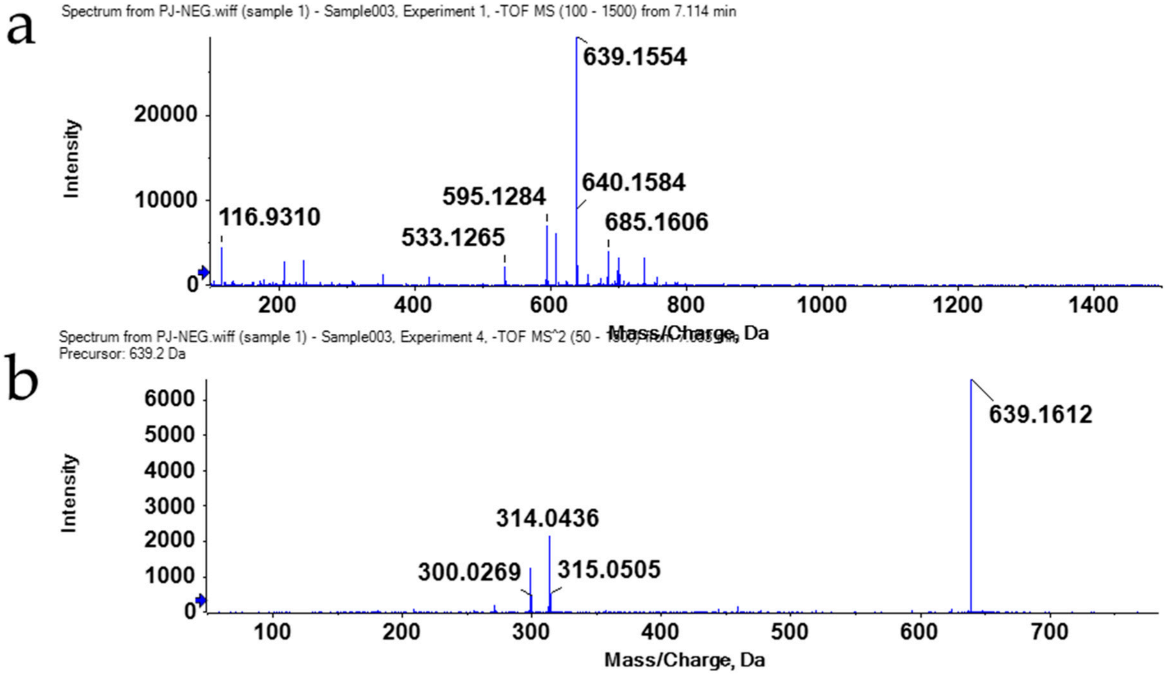

Figure S3. Compound 10 (a) Primary mass spectrum; (b) Secondary mass spectrum.

The peak of Compound 11 was appeared at 6.97 min with the molecular ion at *m*/*z* 595.1284 $[M-H]^-$, and the molecular formula fitted according to the MS^1 results was $C_{26}H_{28}O_{16}$. According to the fragment of 301.0351 $[M-H]^-$ in MS^2 spectra, the presence of glucose structure and xylose structure of the compound was hypothesized. Parent nuclide of the compound is quercetin. After searching of the Scifinder and Reaxys databases, compound 11 was deduced as Quercetin 3-*O*-*β*-*D*-glucopyranosyl-(2->1)-*O*-*β*-*D*-xylopyranoside, the spectra of MS^1, MS^2 and the possible structural formula of this compound are shown in the following figure.

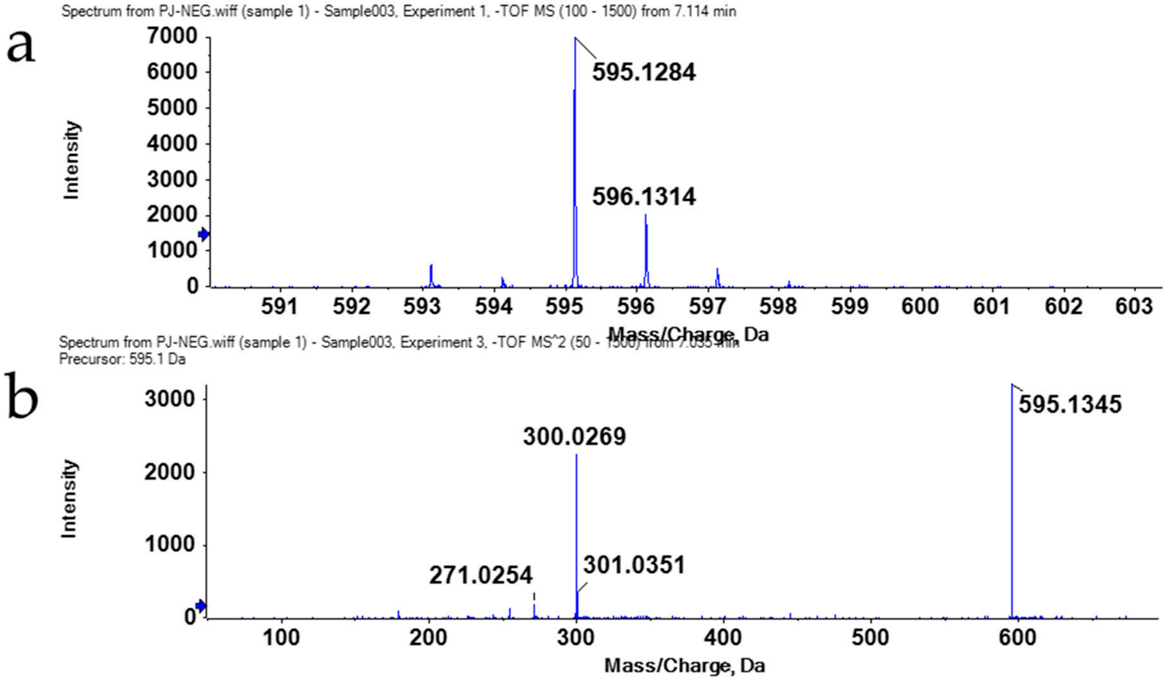

Figure S4. Compound 11 (a) Primary mass spectrum; (b) Secondary mass spectrum.

The peak of Compound 12 was appeared at 12.14 min with the molecular ion at *m*/*z* 533.0919 $[M-H]^-$, and the molecular formula fitted according to the MS^1 results was $C_{24}H_{22}O_{14}$. According to the fragment of 285.0410 $[M-H]^-$ in MS^2 spectra, the presence of glucose structure and malonyl structure of the compound was hypothesized. Parent nuclide of the compound is kaempferol. After searching of the Scifinder and Reaxys databases, compound 12 was deduced as Kaempferol 3-*O*-(6′′-*O*-malonyl) glucoside, the spectra of MS^1, MS^2 and the possible structural formula of this compound are shown in the following figure.

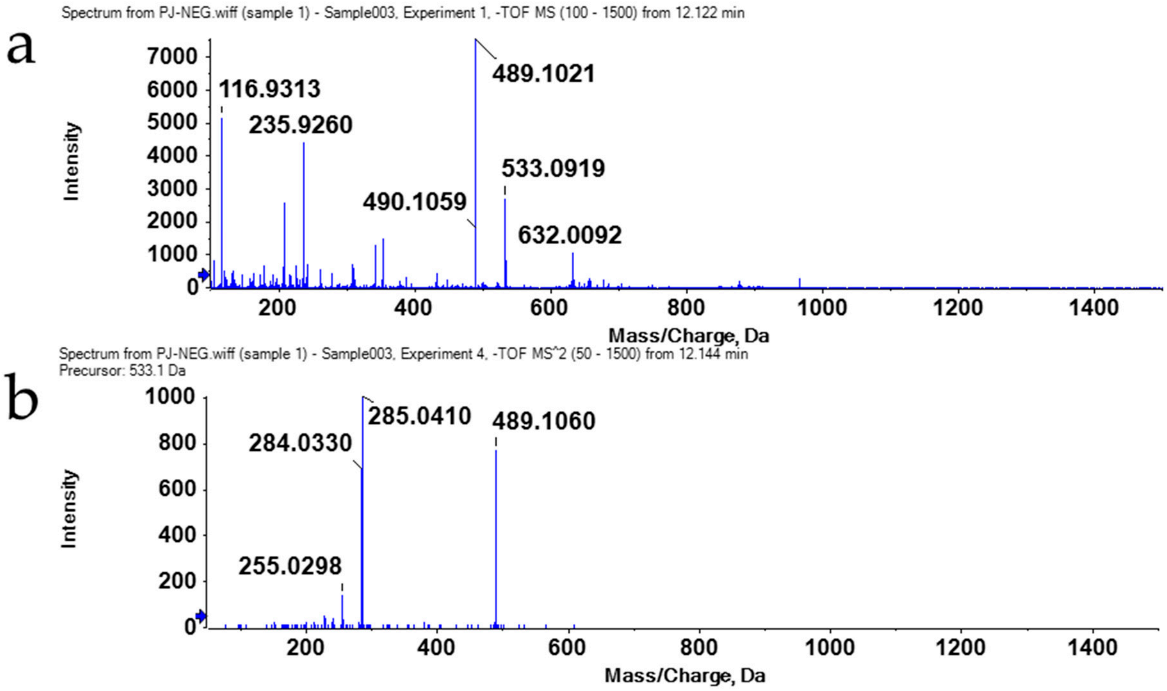

Figure S5. Compound 12 (a) Primary mass spectrum; (b) Secondary mass spectrum.

The peak of Compound 15 was appeared at 8.13 min with the molecular ion at *m/z* 579.1337 $[M-H]^-$, and the molecular formula fitted according to the MS^1 results was $C_{26}H_{28}O_{15}$. According to the fragment of 285.0396 $[M-H]^-$ in MS^2 spectra, the presence of the presence of glucose structure and xylose structure of the compound was hypothesized. Parent nuclide of the compound is kaempferol. After searching of the Scifinder and Reaxys databases, compound 15 was deduced as Kaempferol 3-*O*-*α*-*L*-arabinopyranosyl-(1'''->6'')-*β*-*D*-glucopyranoside, the spectra of MS^1, MS^2 and the possible structural formula of this compound are shown in the following figure.

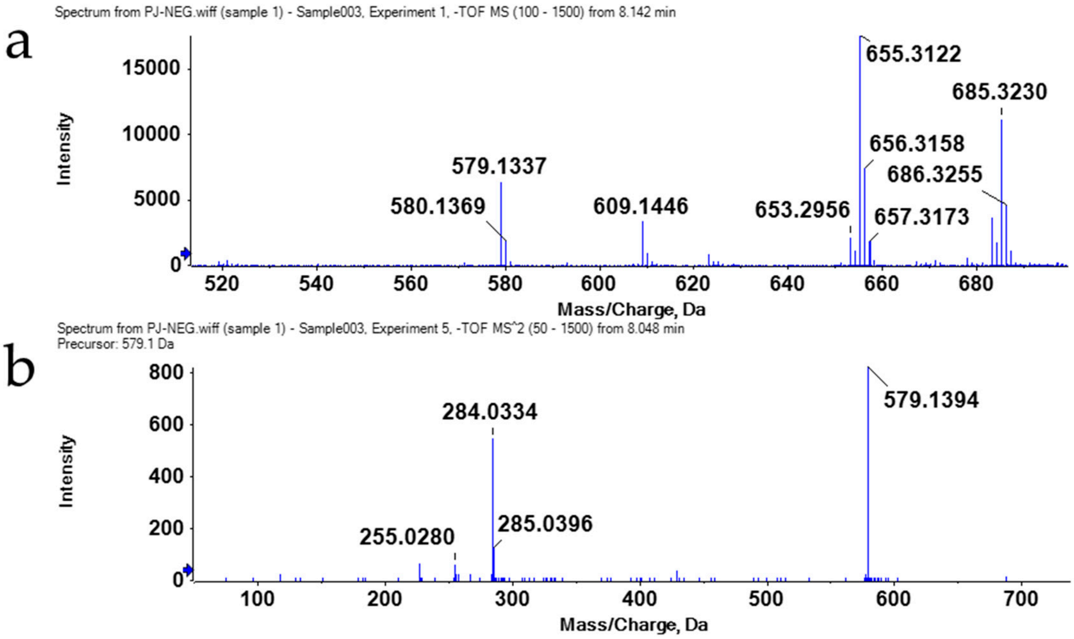

Figure S6. Compound 15 (a) Primary mass spectrum; (b) Secondary mass spectrum.

The peak of Compound 16 was appeared at 8.87 min with the molecular ion at *m/z* 579.1332 $[M-H]^-$, and the molecular formula fitted according to the MS[1] results was $C_{26}H_{28}O_{15}$. According to the fragment of 285.0402 $[M-H]^-$ in MS[2] spectra, the presence of the presence of glucose structure and xylose structure of the compound was hypothesized. Parent nuclide of the compound is kaempferol. After searching of the Scifinder and Reaxys databases, compound 16 was deduced as 3-*O*-[*β*-*D*-xylopyranosyl-(1->2)-*β*-*D*-glucopyranosyl]-kaempferol, the spectra of MS[1], MS[2] and the possible structural formula of this compound are shown in the following figure.

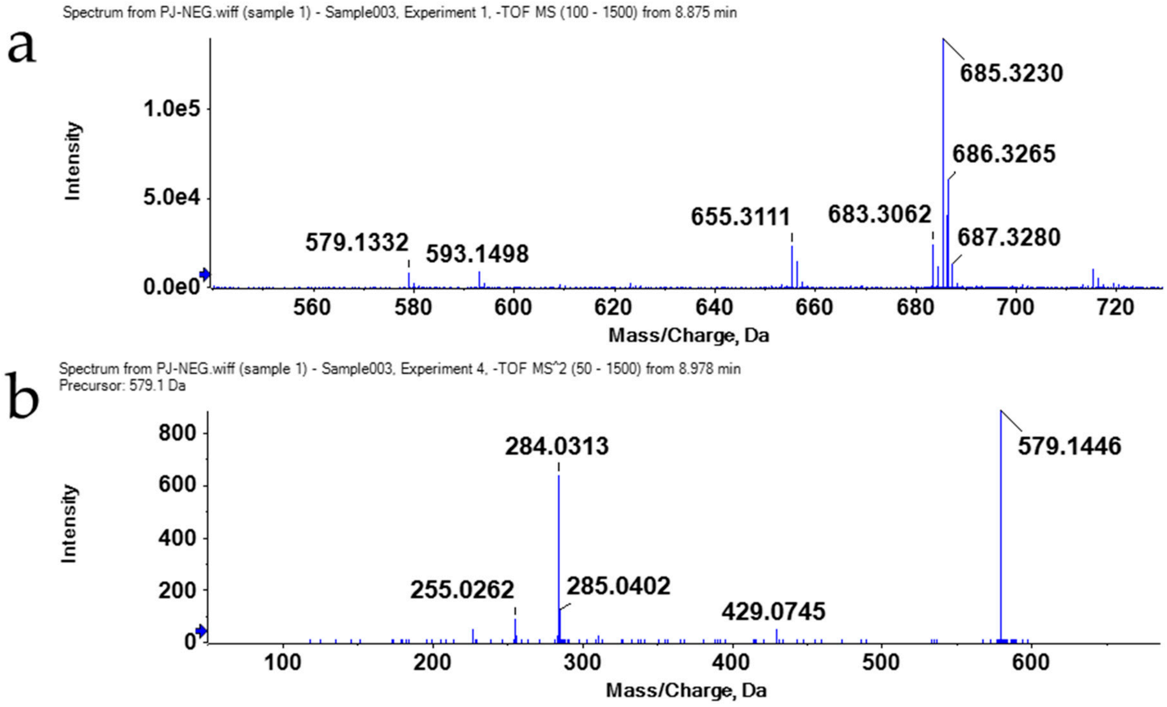

Figure S7. Compound 16 (a) Primary mass spectrum; (b) Secondary mass spectrum.

The peak of Compound 19 was appeared at 9.11 min with the molecular ion at *m/z* 651.1540 $[M-H]^-$, and the molecular formula fitted according to the MS[1] results was $C_{29}H_{32}O_{17}$. According to the fragment of 285.0373 $[M-H]^-$ in MS[2] spectra, the presence of glucose structure and acetyl structure of the compound was hypothesized. Parent nuclide of the compound is kaempferol. After searching of the Scifinder and Reaxys databases, compound 19 was deduced as Kaempferol 3-*O*-*β*-*D*-(2-*O*-*β*-*D*-6-*O*-acetylglucosyl)-glucopyranoside, the spectra of MS[1], MS[2] and the possible structural formula of this compound are shown in the following figure.

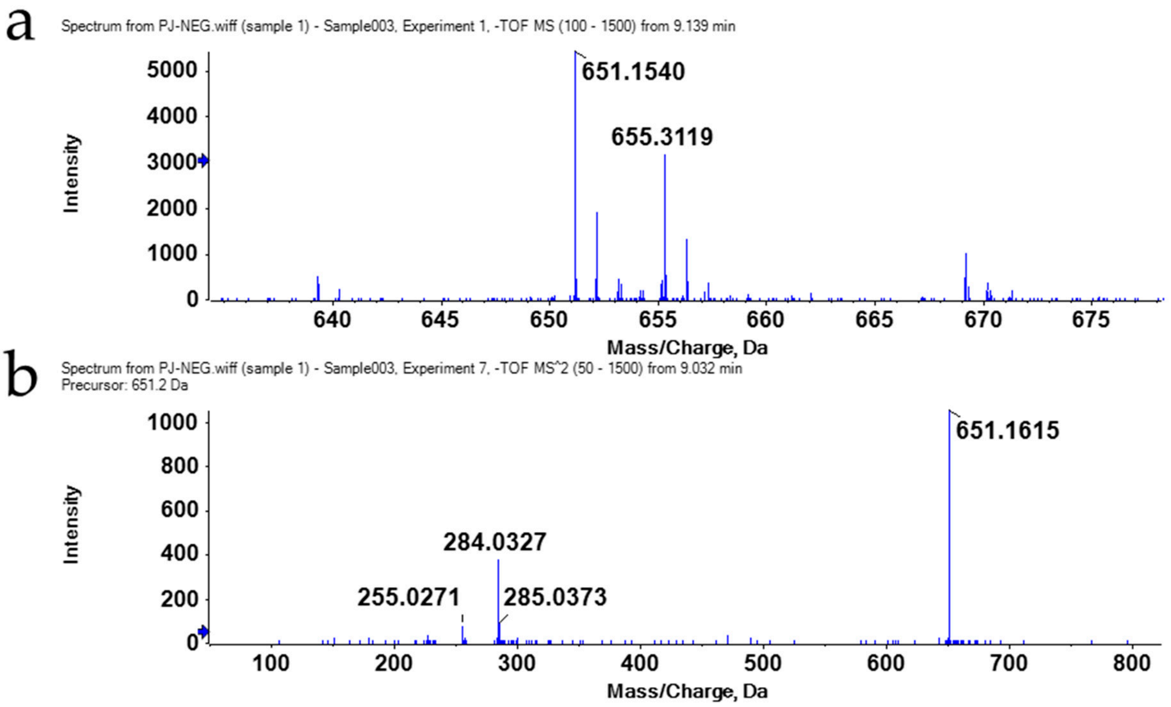

Figure S8. Compound 19 (a) Primary mass spectrum; (b) Secondary mass spectrum.

The peak of Compound 23 peak was appeared at 5.14 min with the molecular ion at *m/z* 436.2228 $[M-H]^-$, and the molecular formula fitted according to the MS^1 results was $C_{25}H_{31}N_3O_4$. According to the fragment of 316.1667 $[M-H-C_8H_7O]^-$, 290.1874 $[M-H-C_9H_5O]^-$, 145.0289 $[C_9H_5O]^-$ and 119.0502 $[C_8H_7O]^-$ in MS^2 spectra, the presence of a caffeoyl structure of the compound was hypothesized. After searching of the Scifinder and Reaxys databases, compound 23 was deduced as N1, N10-bis (p-coumaroyl) spermidine, the spectra of MS^1, MS^2 and the possible structural formula of this compound are shown in the following figure.

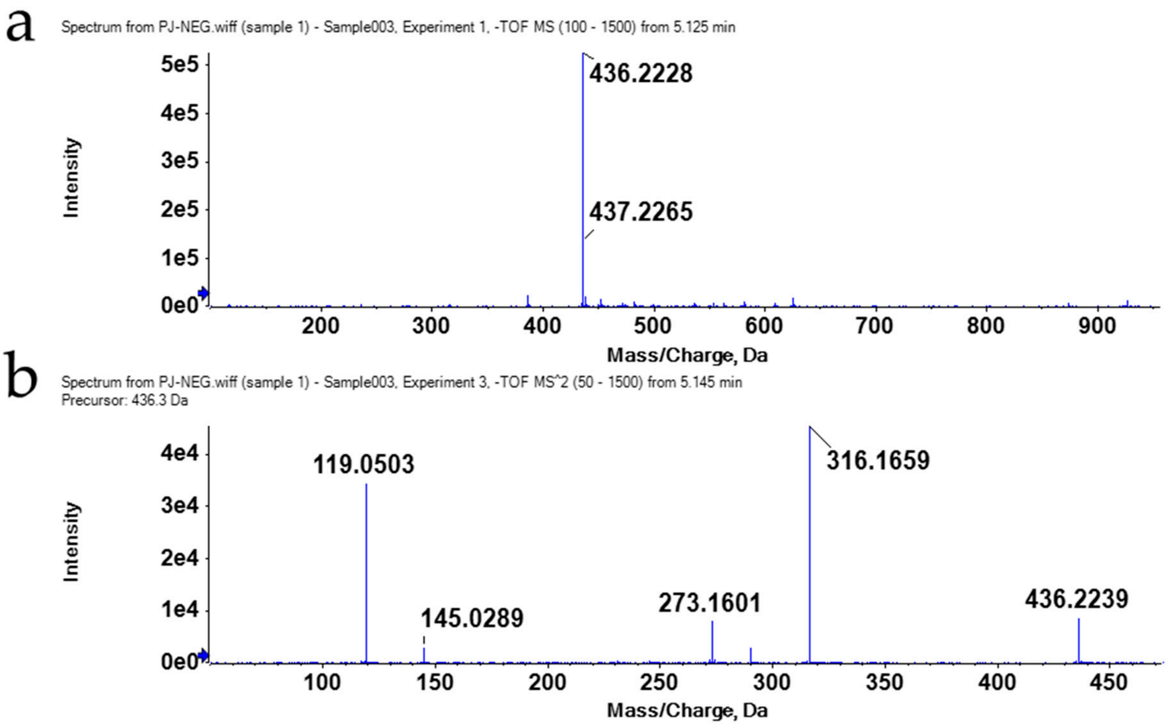

Figure S9. Compound 23 (a) Primary mass spectrum; (b) Secondary mass spectrum.

The peak of Compound 24 peak was appeared at 8.08 min with the molecular ion at *m*/*z* 655.3122 $[M\text{-}H]^-$, and the molecular formula fitted according to the MS^1 results was $C_{37}H_{44}N_4O_7$. According to the fragment of 135.0458 $[C_8H_7O_2]^-$, 399.2071 $[M\text{-}H\text{-}C_8H_7O_2\text{-}C_8H_7O]^-$, 519.2664 $[M\text{-}H\text{-}C_8H_7O_2]^-$ in MS^2 spectra, the presence of a caffeoyl structure of the compound was hypothesized. After searching of the Scifinder and Reaxys databases, compound 24 was deduced as N5, N14-dicoumaroyl-N1-caffeoylspermidine, the spectra of MS^1, MS^2 and the possible structural formula of this compound are shown in the following figure.

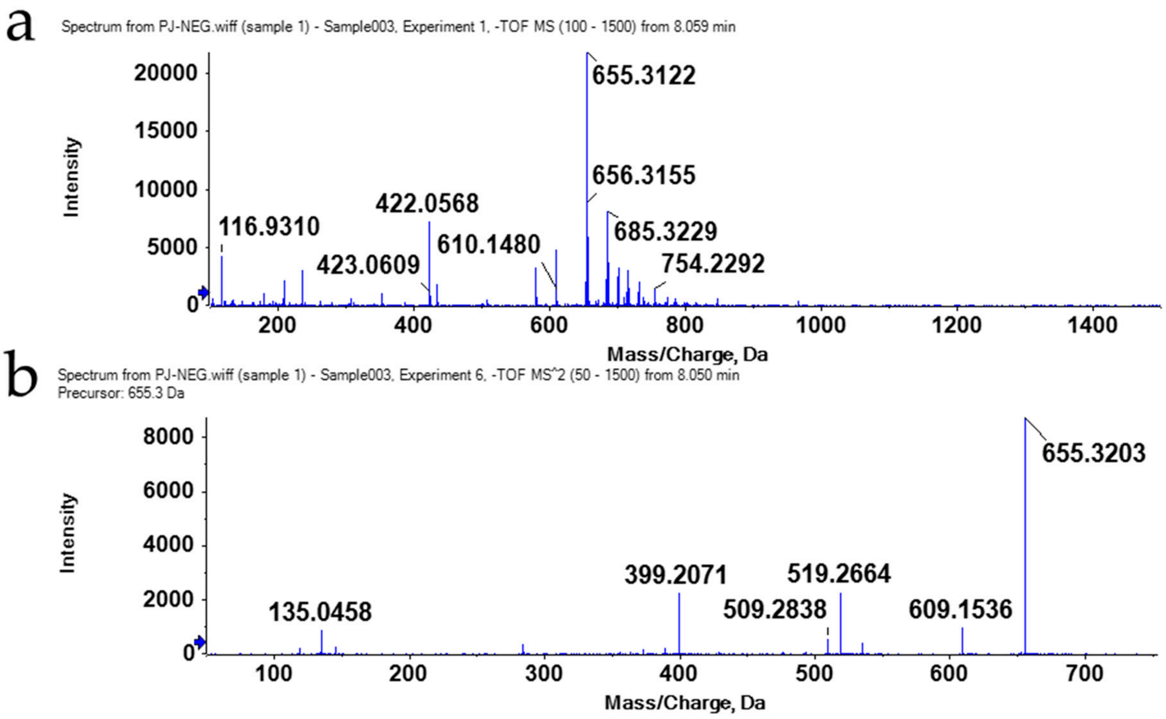

Figure S10. Compound 24 (a) Primary mass spectrum; (b) Secondary mass spectrum.

The peak of Compound 25 peak was appeared at 8.67 min with the molecular ion at *m*/*z* 655.3120 $[M\text{-}H]^-$, and the molecular formula fitted according to the MS[1] results was $C_{37}H_{44}N_4O_7$. According to the fragment of 135.0451 $[C_8H_7O_2]^-$, 399.2063 $[M\text{-}H\text{-}C_8H_7O_2\text{-}C_8H_7O]^-$, 519.2646 $[M\text{-}H\text{-}C_8H_7O_2]^-$ in MS[2] spectra, the presence of a caffeoyl structure of the compound was hypothesized. After searching of the Scifinder and Reaxys databases, compound 26 was deduced as N5, N14-dicoumaroyl-N1-caffeoylspermidine, the spectra of MS[1], MS[2] and the possible structural formula of this compound are shown in the following figure.

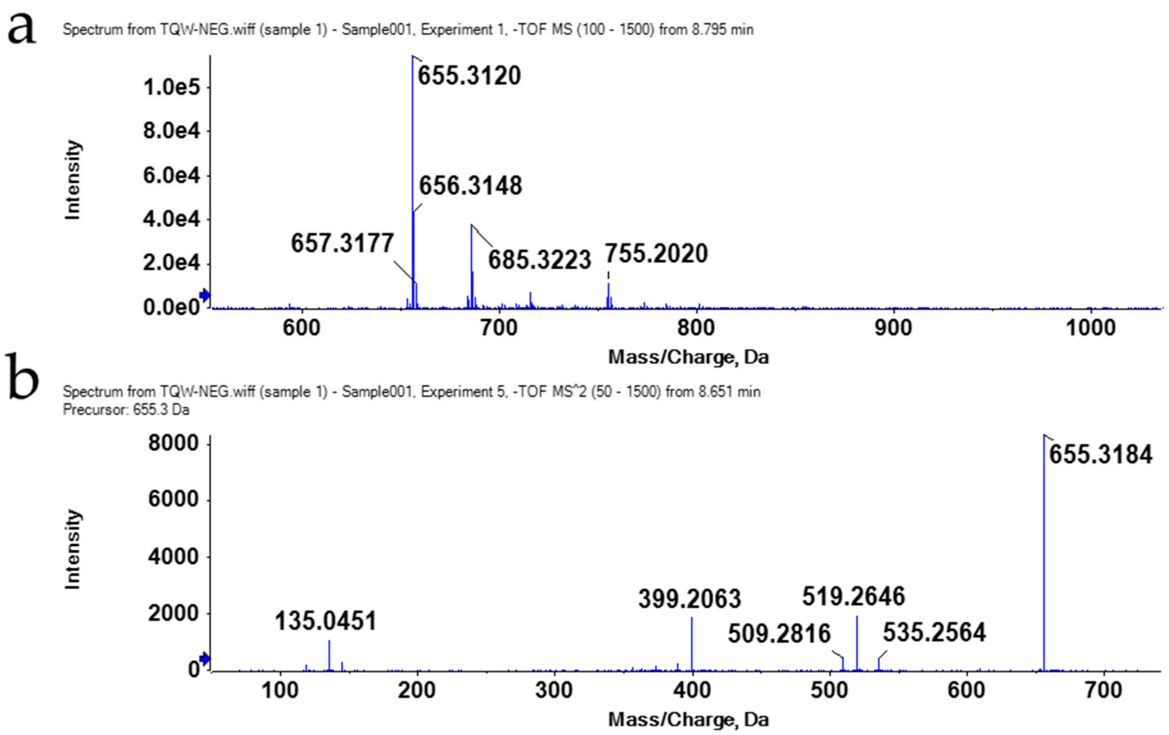

Figure S11. Compound 25 (a) Primary mass spectrum; (b) Secondary mass spectrum.

The peak of Compound 26 peak was appeared at 15.71 min with the molecular ion at *m/z* 582.2594 $[M-H]^-$, and the molecular formula fitted according to the MS^1 results was $C_{34}H_{37}N_3O_6$. According to the fragment of 462.2069 $[M-H-C_8H_7O_2]^-$, 342.1471 $[M-H-C_8H_7O-C_8H_7O]^-$, 145.0300 $[C_9H_5O]^-$, 119.0507 $[C_8H_7O]^-$ in MS^2 spectra, the presence of a p-coumaroyl structure of the compound was hypothesized. After searching of the Scifinder and Reaxys databases, compound 26 was deduced as N1, N5, N10-(Z)-tri-p-coumaroylsper-midine, the spectra of MS^1, MS^2 and the possible structural formula of this compound are shown in the following figure.

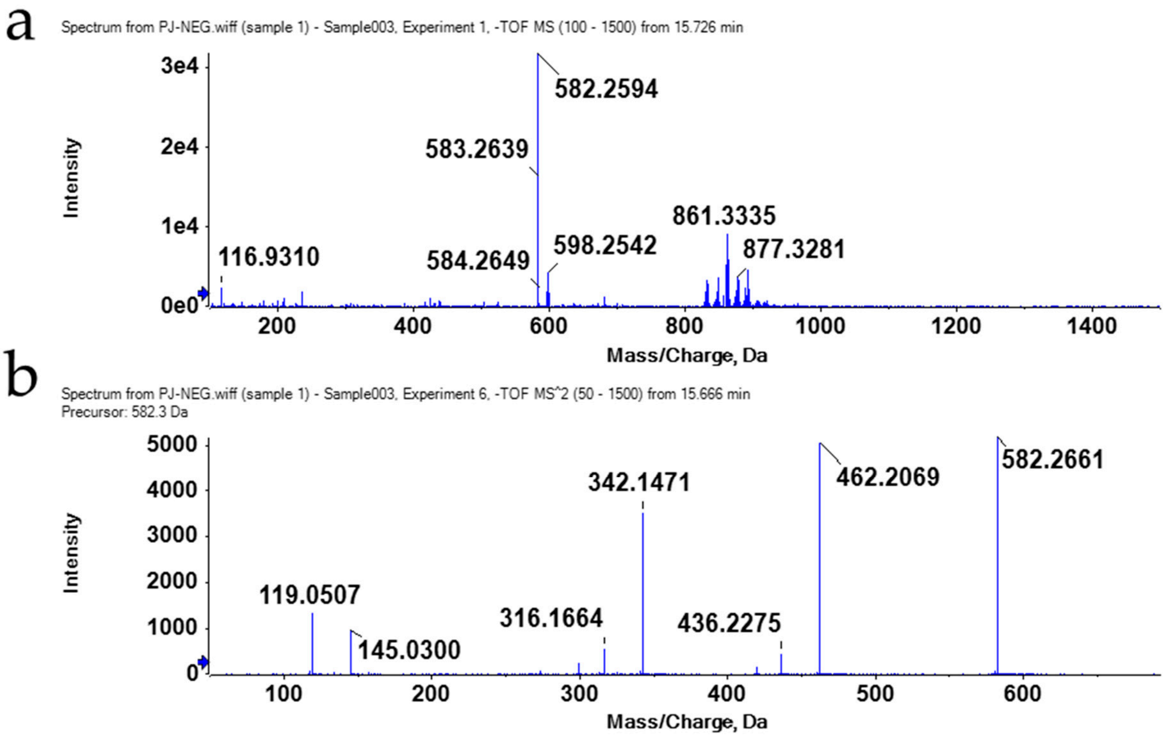

Figure S12. Compound 26 (a) Primary mass spectrum; (b) Secondary mass spectrum.

The peak of Compound 27 peak was appeared at 15.71 min with the molecular ion at *m*/*z* 582.2590 $[M-H]^-$, and the molecular formula fitted according to the MS^1 results was $C_{34}H_{37}N_3O_6$. According to the fragment of 462.2055 $[M-H-C_8H_7O_2]^-$, 342.1465 $[M-H-C_8H_7O-C_8H_7O]^-$, 145.0285 $[C_9H_5O]^-$, 119.0507 $[C_8H_7O]^-$ in MS^2 spectra, the presence of a p-coumaroyl structure of the compound was hypothesized. After searching of the Scifinder and Reaxys databases, compound 27 was deduced as N1, N5-(Z)-N10-(E)-tri-p-coumaroylsper-midine, the spectra of MS^1, MS^2 and the possible structural formula of this compound are shown in the following figure.

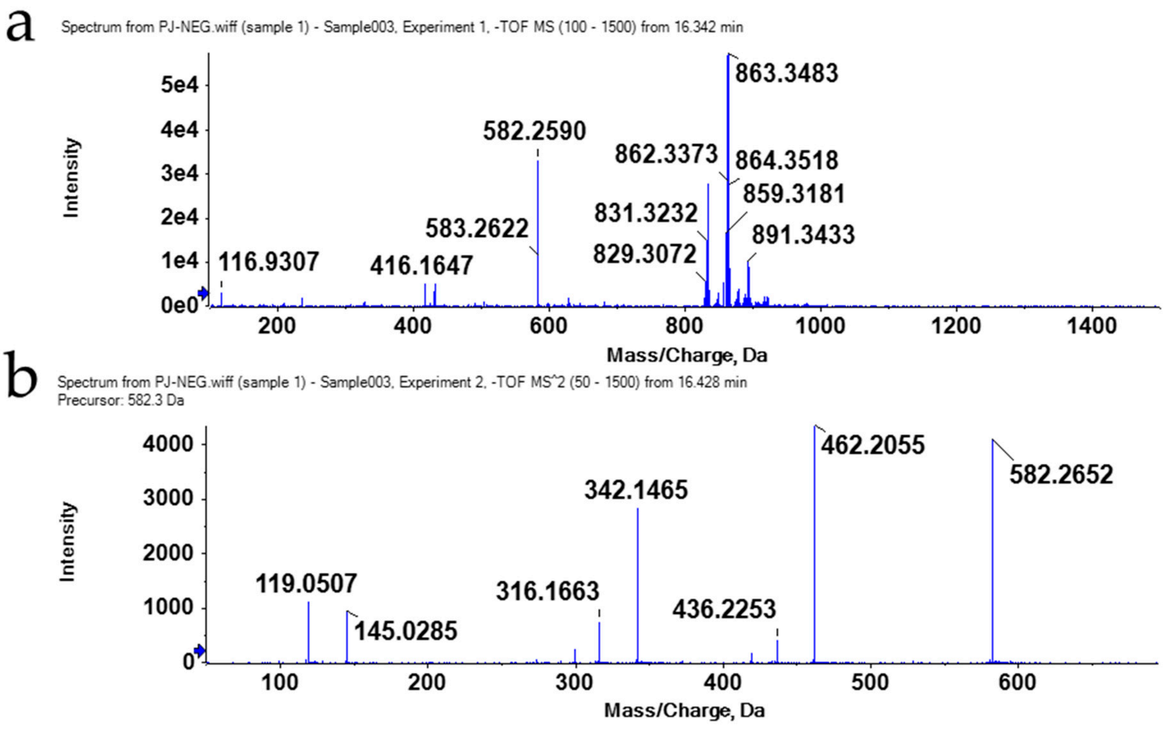

Figure S13. Compound 27 (a) Primary mass spectrum; (b) Secondary mass spectrum.

The peak of Compound 28 was appeared at 16.78 min with the molecular ion at *m*/*z* 582.2589 $[M-H]^-$, and the molecular formula fitted according to the MS^1 results was $C_{34}H_{37}N_3O_6$. According to the fragment of 462.2042 [M-H-C8H7O2]$^-$, 342.1470 [M-H-C8H7O-C8H7O]$^-$, 145.0297 [C9H5O]$^-$, 119.0521 [C8H7O]$^-$ in MS^2 spectra, the presence of a p-coumaroyl structure of the compound was hypothesized. After searching of the Scifinder and Reaxys databases, compound 28 was deduced as N1, N0-(E)-N5-(Z)-tri-p-coumaroylsper-midine, the spectra of MS^1, MS^2 and the possible structural formula of this compound are shown in the following figure.

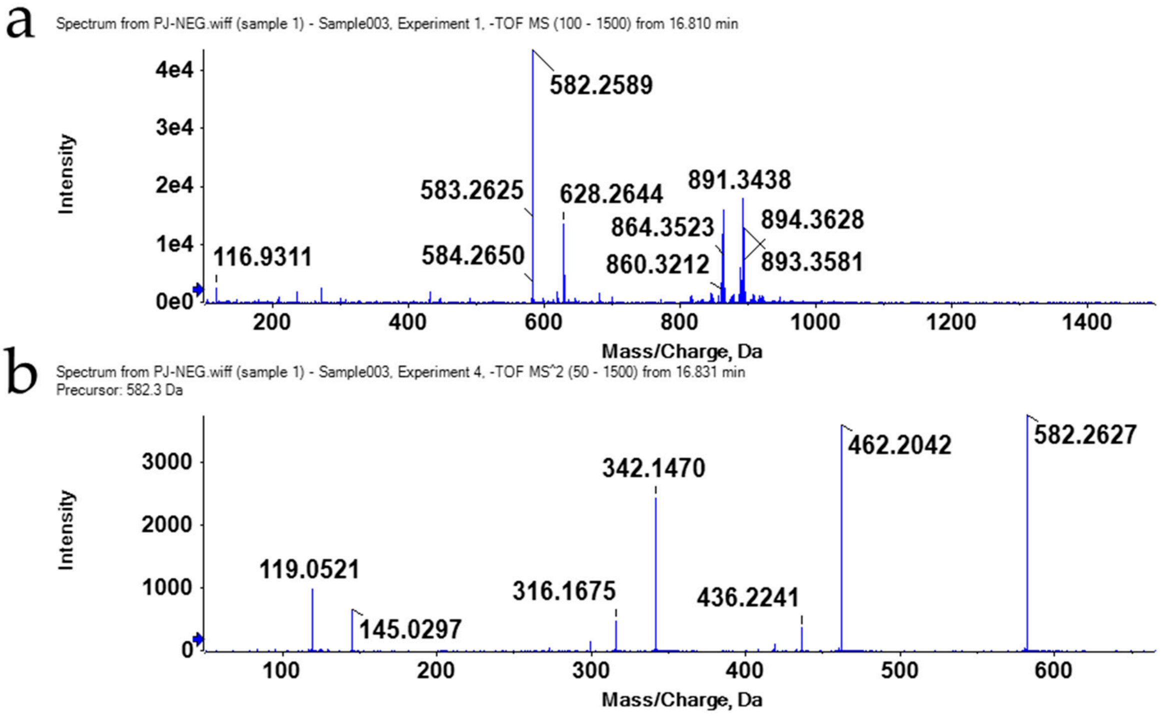

Figure S14. Compound 28 (a) Primary mass spectrum; (b) Secondary mass spectrum.

The peak of Compound 29 was appeared at 17.41 min with the molecular ion at *m*/*z* 582.2593 $[M-H]^-$, and the molecular formula fitted according to the MS^1 results was $C_{34}H_{37}N_3O_6$. According to the fragment of 462.2069 $[M-H-C_8H_7O]^-$, 342.1476 $[M-H-C_8H_7O-C_8H_7O]^-$, 145.0286 $[C_9H_7O_2]^-$, 119.0503 $[C_8H_7O]^-$ in MS^2 spectra, the presence of a p-coumaroyl structure of the compound was hypothesized. After searching of the Scifinder and Reaxys databases, compound 29 was deduced as N1, N5, N10-tri-p-coumaroylspermidine, the spectra of MS^1, MS^2 and the possible structural formula of this compound are shown in the following figure.

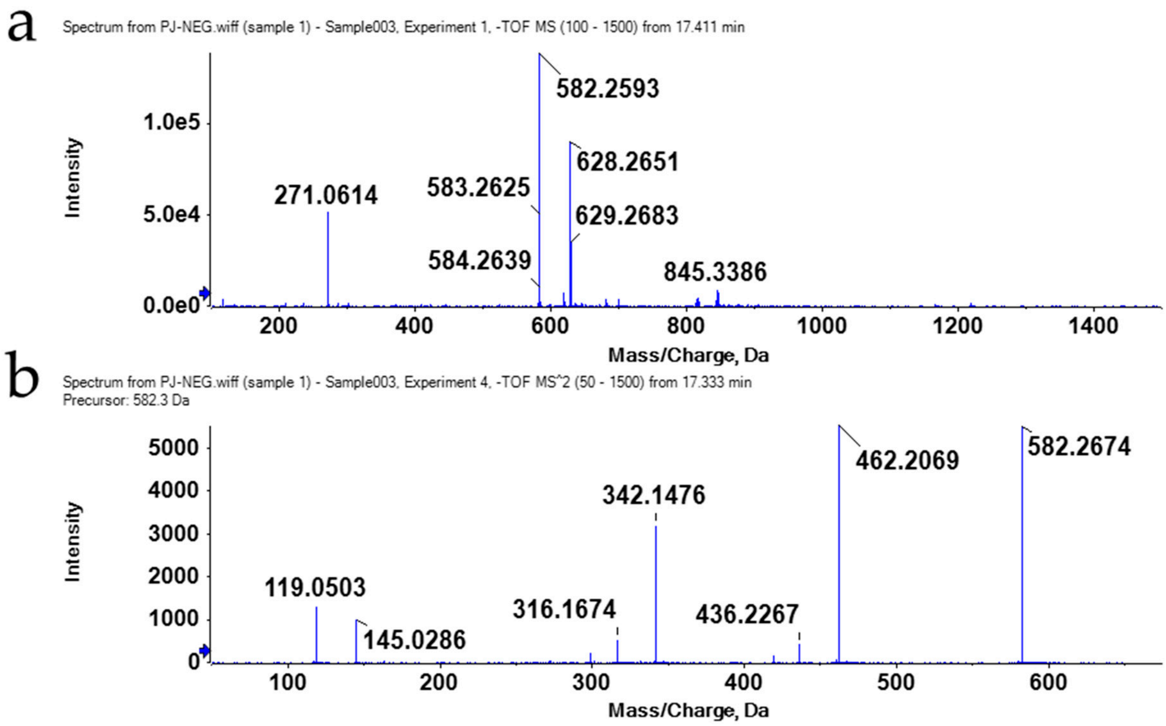

Figure S15. Compound 29 (a) Primary mass spectrum; (b) Secondary mass spectrum.

The peak of Compound 30 was appeared at 17.59 min with the molecular ion at *m/z* 817.3430 $[M-H]^-$, and the molecular formula fitted according to the MS^1 results was $C_{46}H_{50}N_4O_{10}$. According to the fragment of 681.2930 $[M-H-C_8H_7O]^-$, 119.1450 $[C_8H_7O]^-$ in MS^2 spectra, the presence of a p-coumaroyl structure of the compound was hypothesized. After searching of the Scifinder and Reaxys databases, compound 30 was deduced as N5, N10-dicoumaroyl-N1, N14-dicaffeoylspermidine, the spectra of MS^1, MS^2 and the possible structural formula of this compound are shown in the following figure.

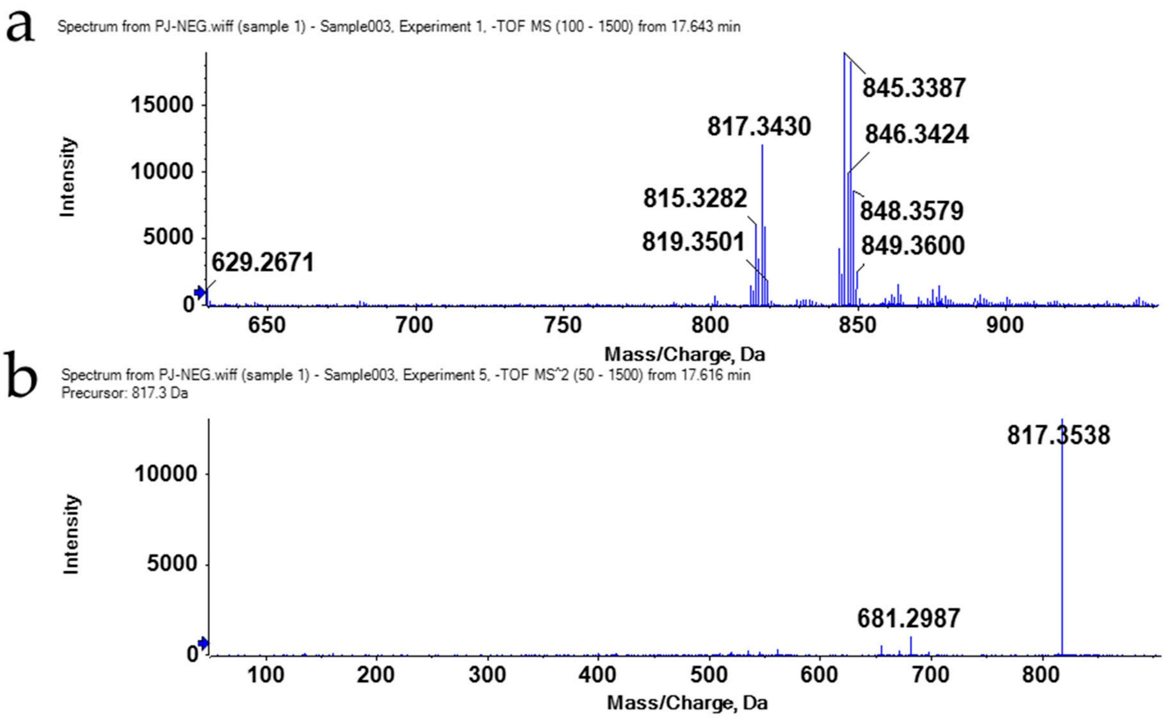

Figure S16. Compound 30. (a) Primary mass spectrum; (b) Secondary mass spectrum.

The peak of Compound 31 was appeared at 16.03 min with the molecular ion at *m/z* 598.2543 $[M-H]^-$, and the molecular formula fitted according to the MS^1 results was $C_{34}H_{37}N_3O_7$. According to the fragment of 478.2012 $[M-H-C_8H_7O]^-$, 316.1662 $[M-H-C_9H_5O_3-C_8H_7O]^-$, 161.0236 $[C_9H_5O_3]^-$, 119.0514 $[C_8H_7O]^-$ in MS^2 spectra, the presence of a p-coumaroyl structure of the compound was hypothesized. After searching of the Scifinder and Reaxys databases, compound 31 was deduced as N10-caffeoyl-N1, N5-di-p-coumaroylsper-midine, the spectra of MS^1, MS^2 and the possible structural formula of this compound are shown in the following figure.

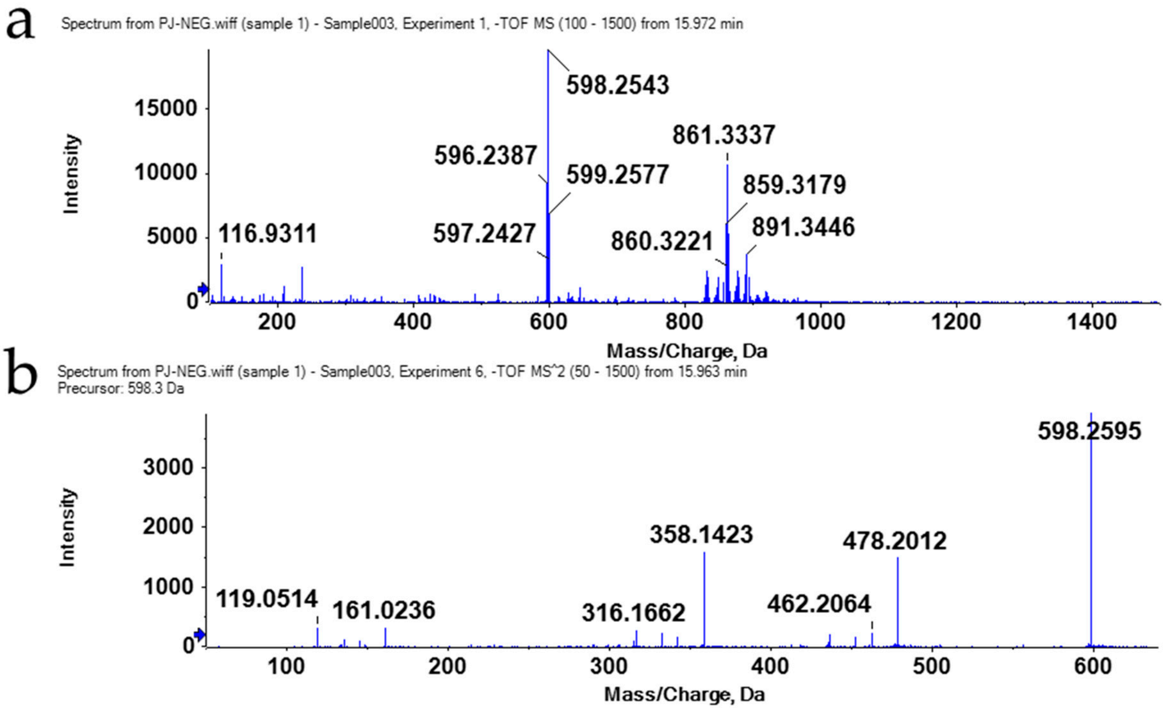

Figure S17. Compound 31. (a) Primary mass spectrum; (b) Secondary mass spectrum.

The peak of Compound 32 was appeared at 18.61 min with the molecular ion at *m/z* 785.3542 $[M\text{-}H]^-$, and the molecular formula fitted according to the MS^1 results was $C_{46}H_{50}N_4O_8$. According to the fragment of 665.3037 $[M\text{-}H\text{-}C_8H_7O]^-$, 545.2448 $[M\text{-}H\text{-}C_6H_5O\text{-}C_9H_7O2]^-$, 145.0292 $[C_9H_7O_2]^-$ in MS^2 spectra, the presence of a p-coumaroyl structure of the compound was hypothesized. After searching of the Scifinder and Reaxys databases, compound 32 was deduced as N1-N5-N10-N14-(Z)-tetra-p-coumaroylspermine, the spectra of MS^1, MS^2 and the possible structural formula of this compound are shown in the following figure.

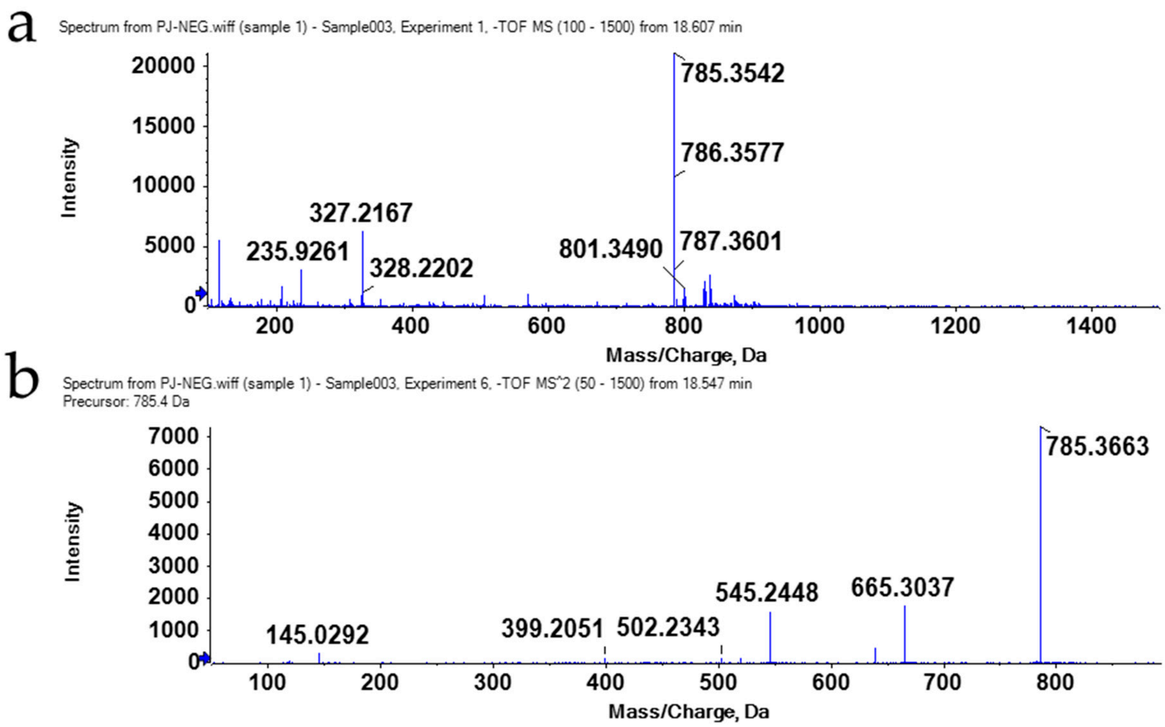

Figure S18. Compound 32. (a) Primary mass spectrum; (b) Secondary mass spectrum.

The peak of Compound 33 was appeared at 19.06 min with the molecular ion at *m*/*z* 785.3544 $[M-H]^-$, and the molecular formula fitted according to the MS[1] results was $C_{46}H_{50}N_4O_8$. According to the fragment of 665.3036 $[M-H-C_8H_7O]^-$, 545.2436 $[M-H-C_6H_5O-C_9H_7O_2]$-, 145.0291 $[C_9H_7O_2]^-$ in MS[2] spectra, the presence of a p-coumaroyl structure of the compound was hypothesized. After searching of the Scifinder and Reaxys databases, compound 33 was deduced as N1-N14-(Z)-N5-N10(E)-tetra-p-coumaroylspermine, the spectra of MS[1], MS[2] and the possible structural formula of this compound are shown in the following figure.

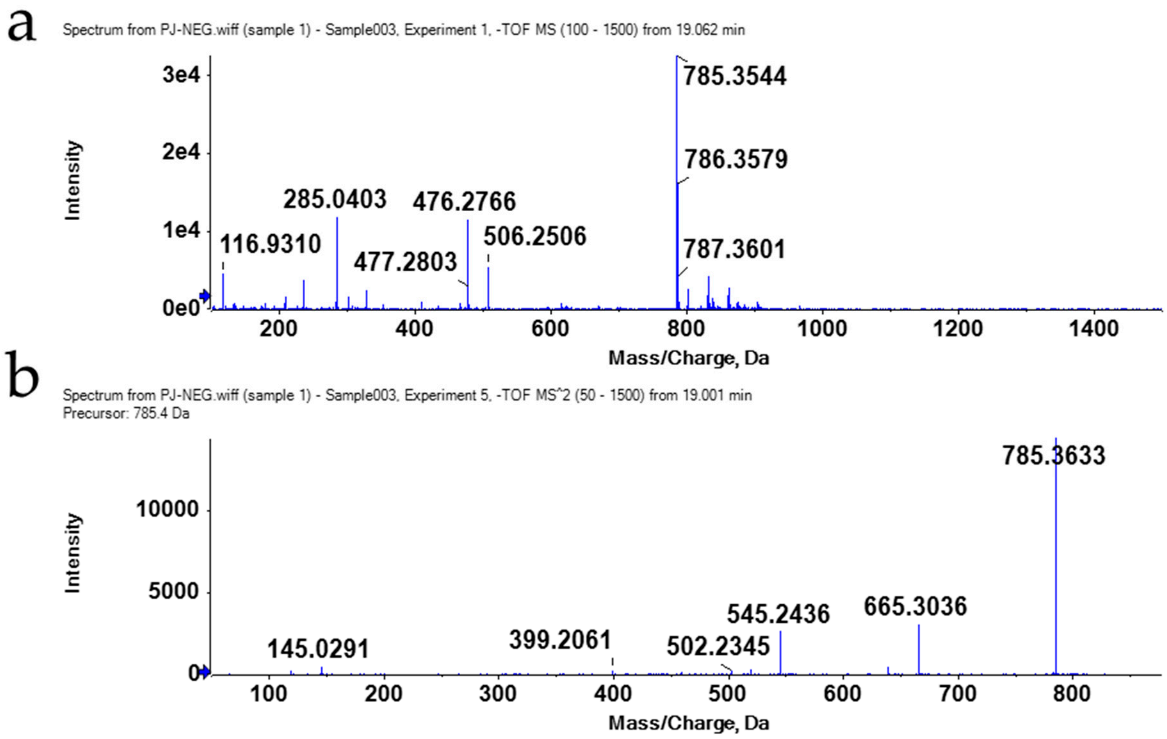

Figure S19. Compound 33. (a) Primary mass spectrum; (b) Secondary mass spectrum.

The peak of Compound 34 was appeared at 19.49 min with the molecular ion at *m*/*z* 785.3540 $[M-H]^-$, and the molecular formula fitted according to the MS^1 results was $C_{46}H_{50}N_4O_8$. According to the fragment of 665.3056 $[M-H-C_8H_7O]^-$, 545.2466$[M-H-C_6H_5O-C_9H_7O_2]$-, 145.0309$[C_9H_7O_2]^-$ in MS^2 spectra, the presence of a p-coumaroyl structure of the compound was hypothesized. After searching of the Scifinder and Reaxys databases, compound 34 was deduced as N1-N14-(E)-N5-N10(Z)-tetra-p-coumaroylspermine, the spectra of MS^1, MS^2 and the possible structural formula of this compound are shown in the following figure.

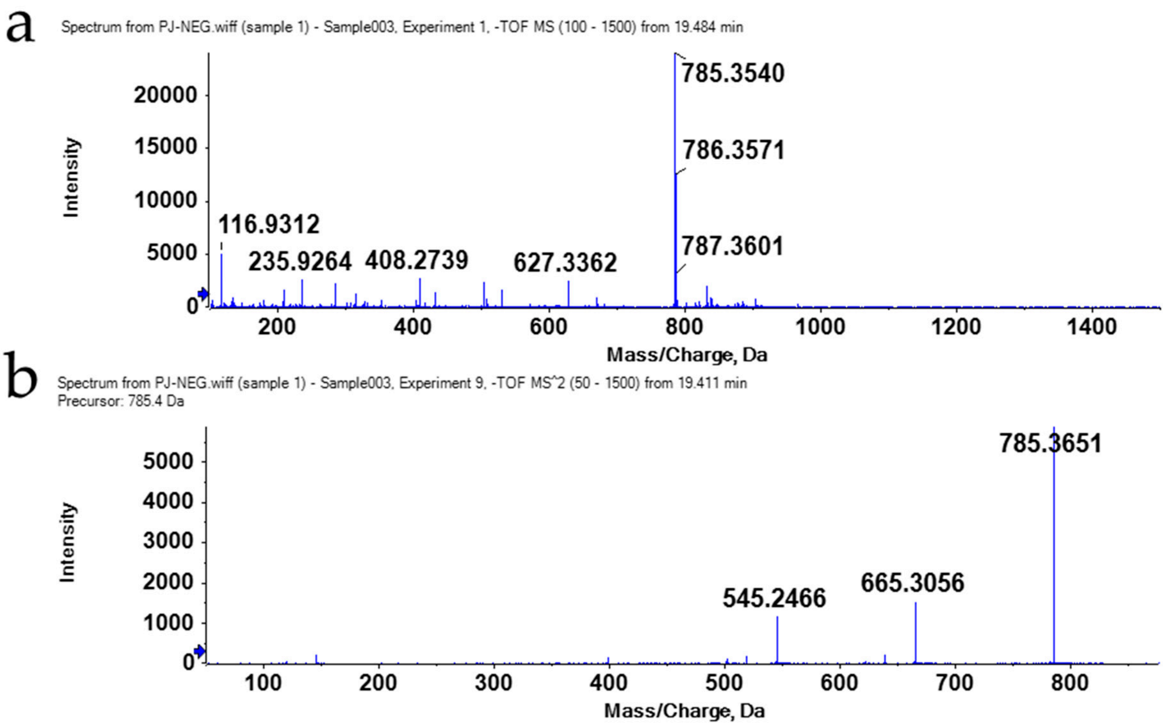

Figure S20. Compound 34. (a) Primary mass spectrum; (b) Secondary mass spectrum.

The peak of Compound 35 was appeared at 20.14 min with the molecular ion at *m*/*z* 785.3543 $[M-H]^-$, and the molecular formula fitted according to the MS^1 results was $C_{46}H_{50}N_4O_8$. According to the fragment of 665.3042$[M-H-C_8H_7O]^-$, 545.2438$[M-H-C_6H_5O-C_9H_7O_2]^-$, 145.0286 $[C_9H_7O_2]^-$ in MS^2 spectra, the presence of a p-coumaroyl structure of the compound was hypothesized. After searching of the Scifinder and Reaxys databases, compound 35 was deduced as N1, N5, N10, N14-tetra-p-coumaroylspermine, the spectra of MS^1, MS^2 and the possible structural formula of this compound are shown in the following figure.

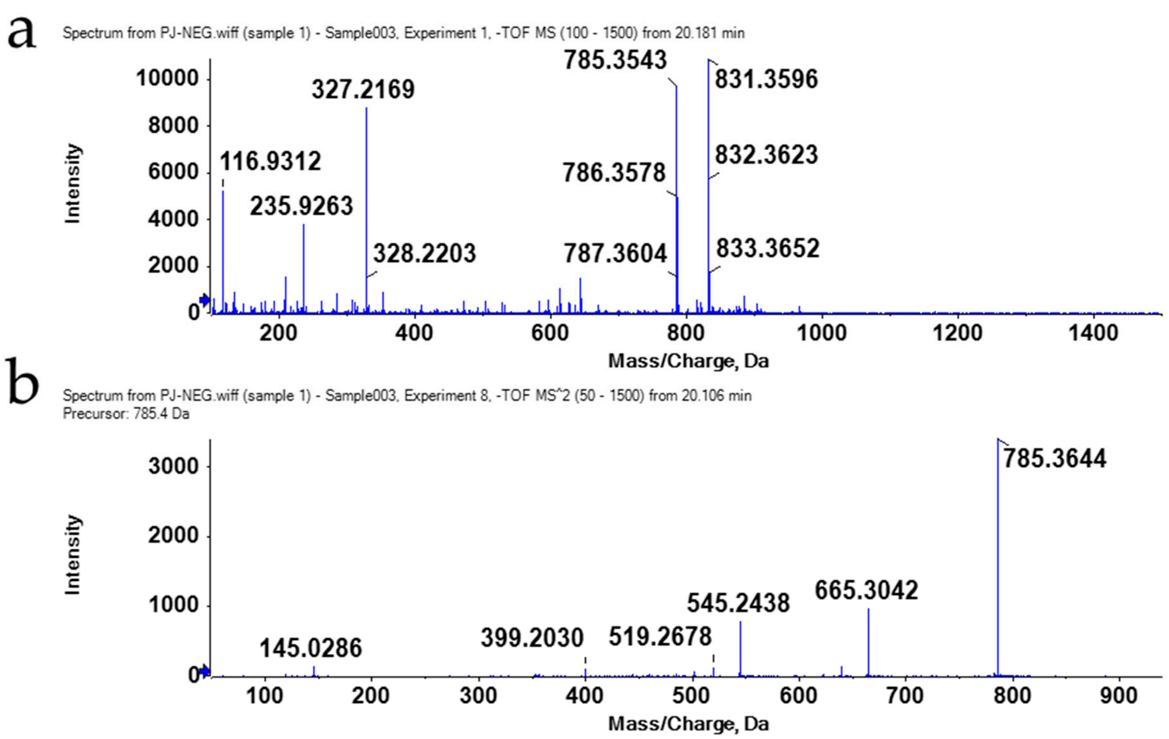

Figure S21. Compound 35. (a) Primary mass spectrum; (b) Secondary mass spectrum.

The peak of Compound 36 was appeared at 12.95 min with the molecular ion at *m*/*z* 630.2441 $[M-H]^-$, and the molecular formula fitted according to the MS[1] results was $C_{34}H_{37}N_3O_9$. According to the fragment of 494.1946 $[M-H-C_8H_7O_2]^-$, 468.2174 $[M-H-C_9H_7O_3]^-$, 358.1423 $[M-H-C_9H_7O_3-C_6H_5O_2]^-$, 161.0240$[C_9H_5O_3]^-$, 135.0441$[C_8H_7O_2]^-$in MS[2] spectra, the presence of a caffeoyl structure of the compound was hypothesized. After searching of the Scifinder and Reaxys databases, compound 36 was deduced as N1, N5, N10-(E)-tricaffeoylspermidine, the spectra of MS[1], MS[2] and the possible structural formula of this compound are shown in the following figure.

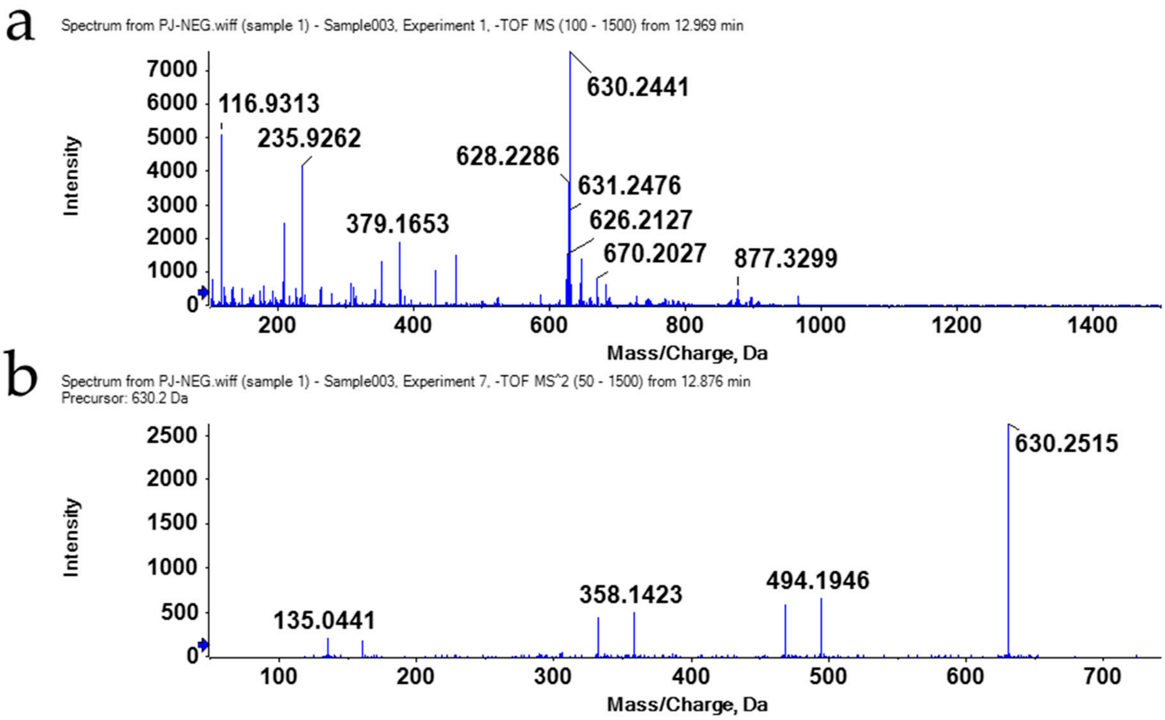

Figure S22. Compound 36. (a) Primary mass spectrum; (b) Secondary mass spectrum.

The peak of Compound 37 was appeared at 12.85 min with the molecular ion at *m/z* 379.1653 $[M-H]^-$, and the molecular formula fitted according to the MS^1 results was $C_{22}H_{24}N_2O_4$. According to the fragment of 259.1069 $[M-H-C_8H_7O]^-$, 145.0295 $[C_9H_5O_2]^-$, 119.0502 $[C_8H_7O]^-$ In MS^2 spectra, the presence of a caffeoyl structure structure of the compound was hypothesized. After searching of the Scifinder and Reaxys databases, compound 37 was deduced as N, N'-di (coumaroyl) putrescine, the spectra of MS^1, MS^2 and the possible structural formula of this compound are shown in the following figure.

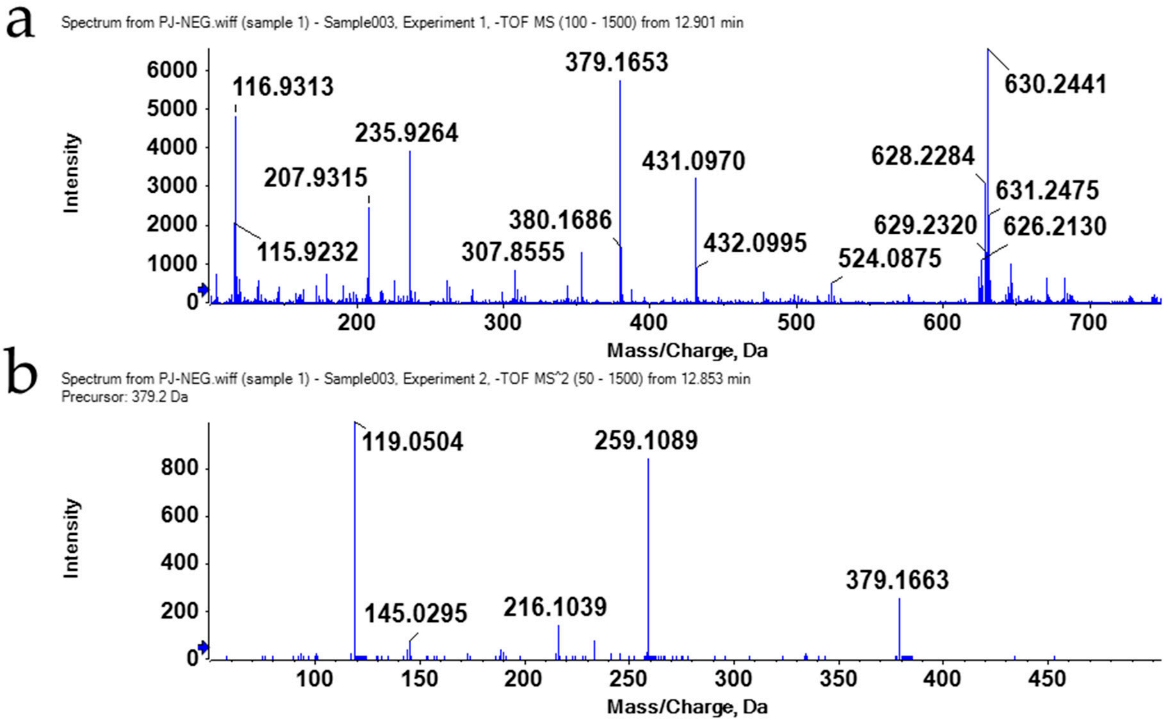

Figure S23. Compound 37. (a) Primary mass spectrum; (b) Secondary mass spectrum.

The peak of Compound 38 was appeared at 4.12 min with the molecular ion at *m*/*z* 778.5345 $[M-H]^+$, and the molecular formula fitted according to the MS[1] results was $C_{44}H_{76}NO_8P$. According to the fragment of 184.0714 $[M-H]^+$ in MS[2] spectra, the compound is deduced to be phospholipids. After searching of the Scifinder and Reaxys databases, compound 38 was deduced as 1,2-Dilinolenoyl-sn-glycero-3-phosphocholine, the spectra of MS[1], MS[2] and the possible structural formula of this compound are shown in the following figure.

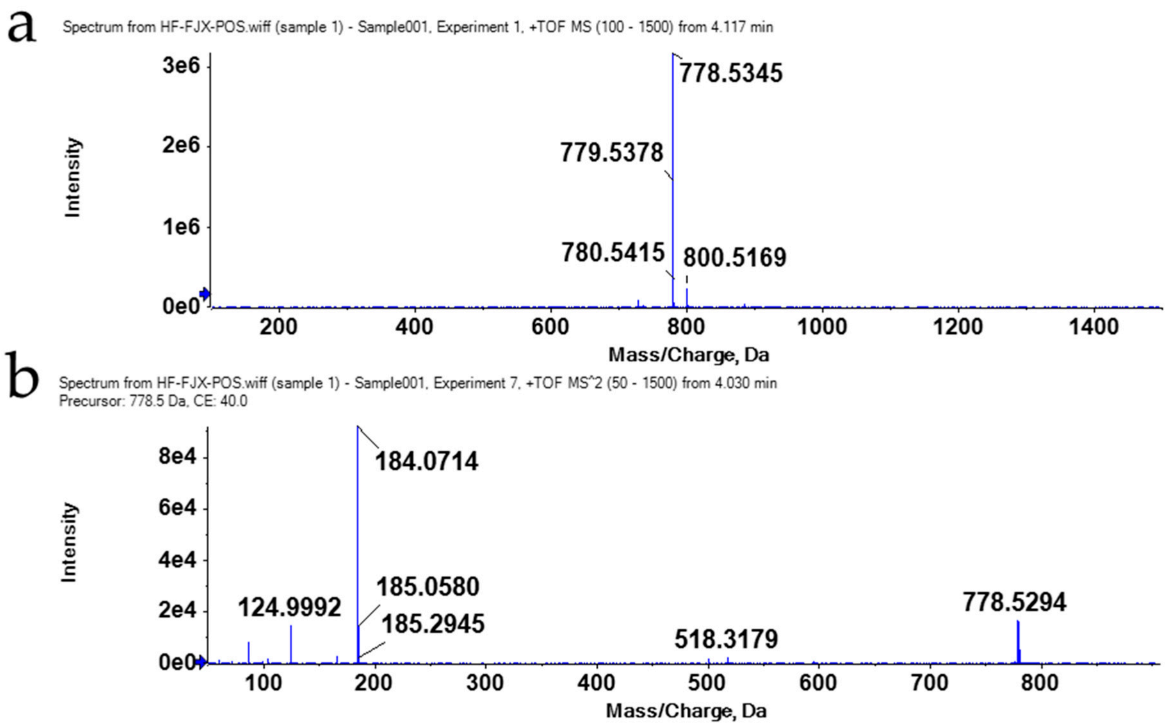

Figure S24. Compound 38. (a) Primary mass spectrum; (b) Secondary mass spectrum.

The peak of Compound 39 was appeared at 4.52 min with the molecular ion at *m/z* 780.5506 $[M-H]^+$, and the molecular formula fitted according to the MS^1 results was $C_{44}H_{78}NO_8P$. According to the fragment of 184.0728 $[M-H]^+$ in MS^2 spectra, the compound is deduced to be phospholipids. After searching of the Scifinder and Reaxys databases, compound 39 was deduced as PC-C18:3/C18:2, the spectra of MS^1, MS^2 and the possible structural formula of this compound are shown in the following figure.

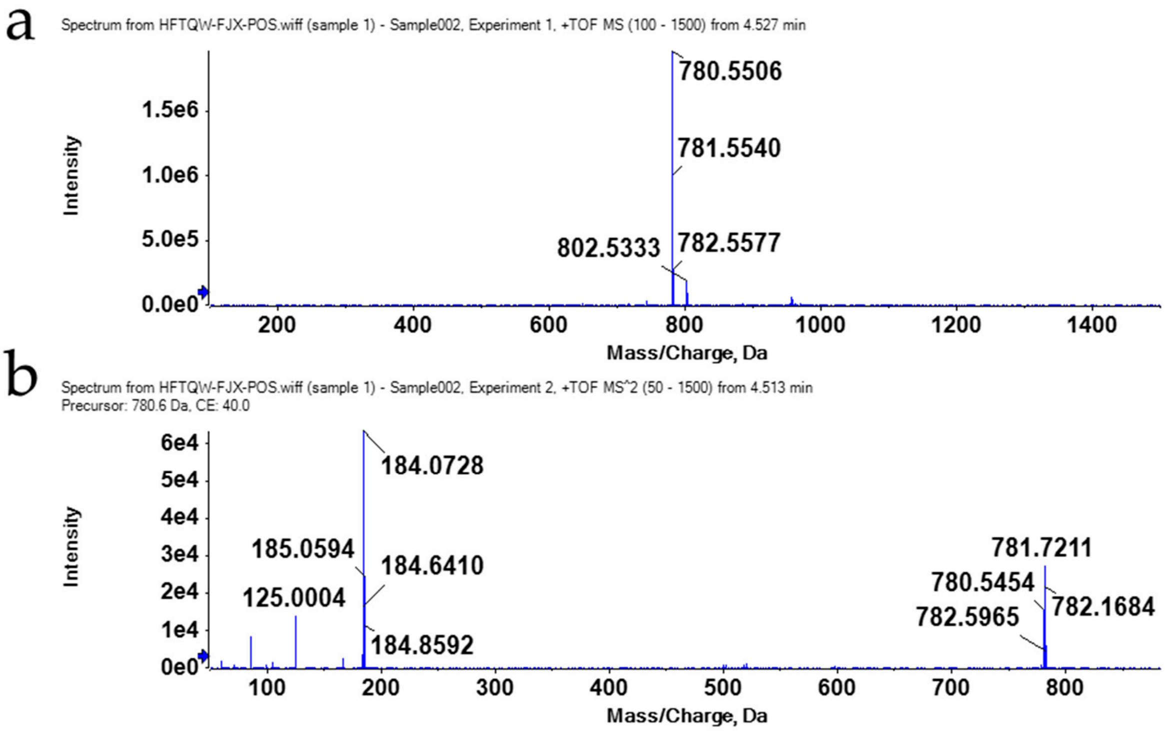

Figure S25. Compound 39. (a) Primary mass spectrum; (b) Secondary mass spectrum.

The peak of Compound 40 was appeared at 4.88 min with the molecular ion at *m/z* 756.5507 $[M-H]^+$, and the molecular formula fitted according to the MS^1 results was $C_{44}H_{78}NO_8P$. According to the fragment of 184.0719 $[M-H]^+$ in MS^2 spectra, the compound is deduced to be phospholipids. After searching of the Scifinder and Reaxys databases, compound 40 was deduced as 1-Palmitoyl-2-linoleoyl-3-sn-phosphatidylcholine, the spectra of MS^1, MS^2 and the possible structural formula of this compound are shown in the following figure.

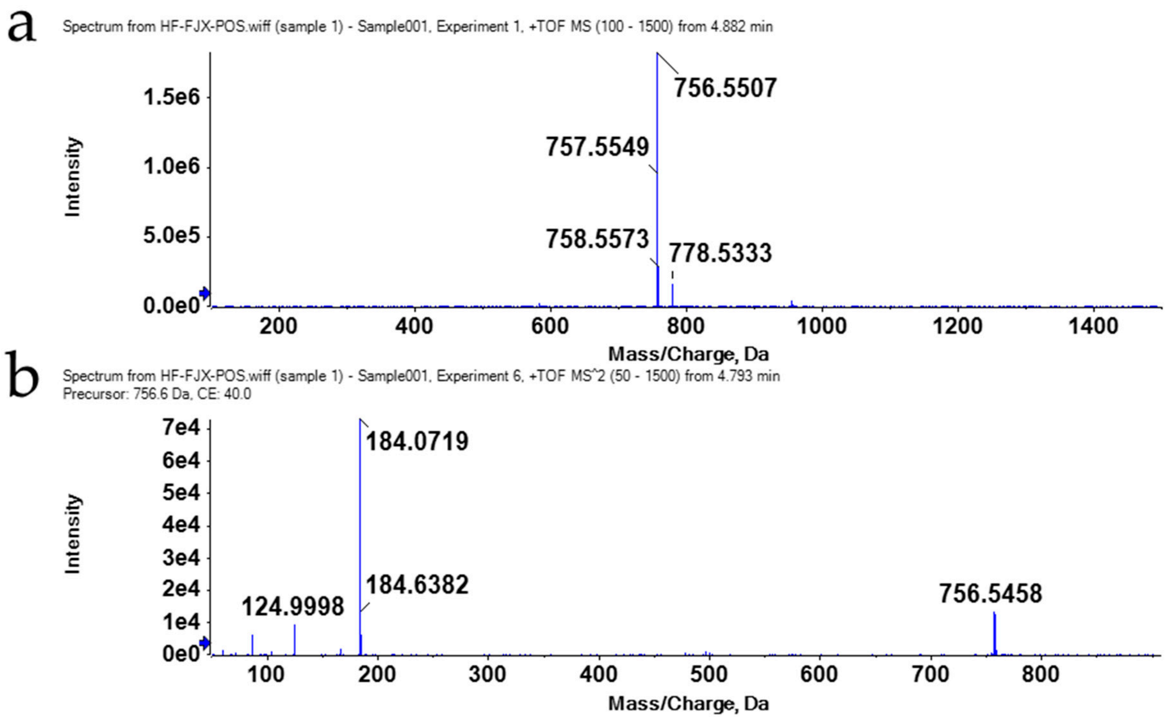

Figure S26. Compound 40. (a) Primary mass spectrum; (b) Secondary mass spectrum.

The peak of Compound 41 was appeared at 5.04 min with the molecular ion at m/z 782.5666 $[M-H]^+$, and the molecular formula fitted according to the MS1 results was $C_{44}H_{80}NO_8P$. According to the fragment of 184.0736 $[M-H]^+$ in MS^2 spectra, the compound is deduced to be phospholipids. After searching of the Scifinder and Reaxys databases, compound 41 was deduced as 1,2-Dilinoleoyl-sn-glycero-3-phosphocholine, the spectra of MS^1, MS2 and the possible structural formula of this compound are shown in the following figure.

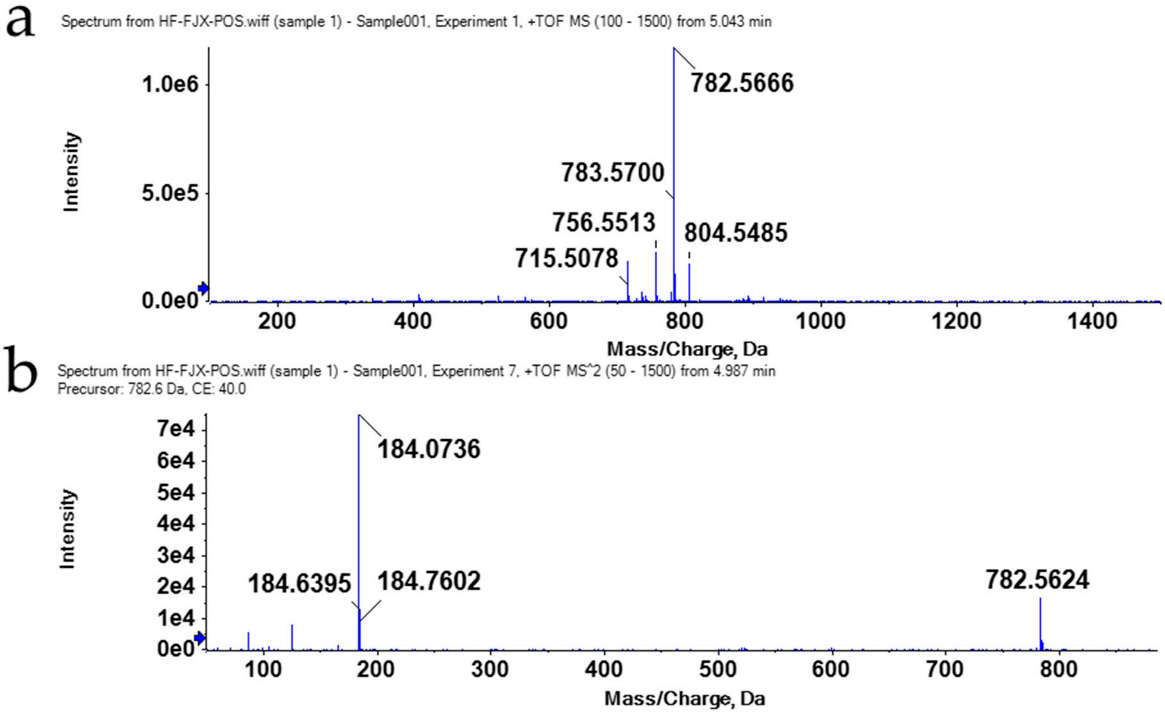

Figure S27. Compound 41. (a) Primary mass spectrum; (b) Secondary mass spectrum.

The peak of Compound 49 was appeared at 6.60 min with the molecular ion at *m*/*z* 388.0386 $[M-H]^-$, and the molecular formula fitted according to the MS^1 results was $C_{11}H_{19}NO_{10}S_2$. According to the fragment of 96.9625 $[M-H]^+$ in MS^2 spectra, the compound is deduced to be phospholipids. After searching of the Scifinder and Reaxys databases, compound 49 was deduced as 1,2-Dilinoleoyl-sn-glycero-3-phosphocholine, the spectra of MS^1, MS^2 and the possible structural formula of this compound are shown in the following figure.

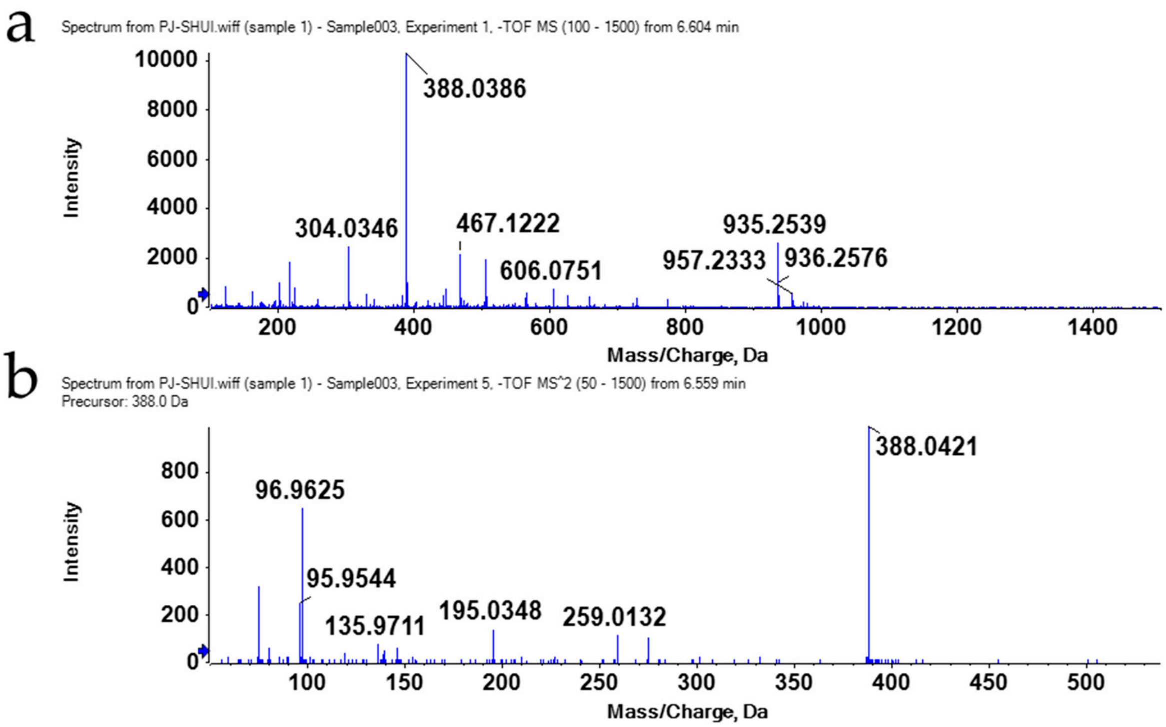

Figure S28. Compound 49. (a) Primary mass spectrum; (b) Secondary mass spectrum.
